# Activity of CK2α protein kinase is required for efficient replication of some HPV types

**DOI:** 10.1371/journal.ppat.1007788

**Published:** 2019-05-15

**Authors:** Alla Piirsoo, Marko Piirsoo, Martin Kala, Eve Sankovski, Elina Lototskaja, Viktor Levin, Mauro Salvi, Mart Ustav

**Affiliations:** 1 Institute of Technology, University of Tartu, Tartu, Estonia; 2 Department of Biomedical Sciences, University of Padova, Padova, Italy; University of Wisconsin Madison School of Medicine and Public Health, UNITED STATES

## Abstract

Inhibition of human papillomavirus (HPV) replication is a promising therapeutic approach for intervening with HPV-related pathologies. Primary targets for interference are two viral proteins, E1 and E2, which are required for HPV replication. Both E1 and E2 are phosphoproteins; thus, the protein kinases that phosphorylate them might represent secondary targets to achieve inhibition of HPV replication. In the present study, we show that CX4945, an ATP-competitive small molecule inhibitor of casein kinase 2 (CK2) catalytic activity, suppresses replication of different HPV types, including novel HPV5NLuc, HPV11NLuc and HPV18NLuc marker genomes, but enhances the replication of HPV16 and HPV31. We further corroborate our findings using short interfering RNA (siRNA)-mediated knockdown of CK2 α and α’ subunits in U2OS and CIN612 cells; we show that while both subunits are expressed in these cell lines, CK2α is required for HPV replication, but CK2α’ is not. Furthermore, we demonstrate that CK2α acts in a kinase activity-dependent manner and regulates the stability and nuclear retention of endogenous E1 proteins of HPV11 and HPV18. This unique feature of CK2α makes it an attractive target for developing antiviral agents.

## Author summary

Human papillomaviruses (HPVs) are small DNA viruses that infect epithelial cells and can cause a variety of hyperplastic changes in the infected tissue. Replication of the HPV genomes relies largely on the host cell factors. The only viral proteins that are necessary for the virus replication are E1 and E2. Relatively little is known about the host cell factors that modulate the activities of E1 and E2 proteins and thereby regulate replication of the HPV genome. We have discovered that the activity of an ubiquitously expressed protein kinase CK2 is needed for efficient replication of a number of different HPV types. We further show that only one of the two catalytic subunits of CK2 existing in the cell, CK2α, is required for the replication of HPV genomes, whereas a closely related CK2α’ has no or little effect. Finally, we demonstrate that inhibition of CK2 activity results in rapid degradation of the E1 helicase and thereby inefficient replication of the HPV genomes. Taken together our results delineate the function of a host cell protein kinase CK2 in the HPV life cycle and describe a surprising differential effect of two closely related catalytic subunits of CK2 in the replication of HPV genomes.

## Introduction

Human papillomaviruses (HPVs) infect cutaneous and mucosal basal epithelial cells retaining an important position among sexually transmitted infections worldwide. Although the majority of HPV infections are transient, persistent infection with high-risk (HR) HPVs may instigate cellular transformation and cancer, whereas infection with low-risk (LR) HPVs induces benign tissue changes and warts [1]. At least 15 different virus types belong to HR HPVs contributing to the pathogenesis of virtually all cases of cervical cancer and a subset of other epithelial tumors such as head and neck cancers [2][3].

Although vaccination against the most prevalent HPV types is available, cervical cancer is also associated with HPV subtypes not covered by the vaccines [4]. Additionally, therapeutic strategies targeting already existing HPV infections harbored by up to 20% of the population are required to prevent and alleviate HPV-derived pathologies. The design and applications of antiviral drugs depend on thorough understanding of the HPV infection cycle and its relationship with host cells.

The HPV infection cycle is strictly dependent on the epithelial differentiation program. Infection begins with intrusion of HPV into mitotically active basal epithelial stem cells, followed by virus genome uncoating, transport to the nucleus and expression of viral early genes E1 and E2. E1 and E2 encode the only viral proteins required for HPV genome replication throughout all phases of the virus life cycle. E1 is an ATP-dependent DNA helicase that forms a double-hexamer in its enzymatically active form and unwinds DNA, whereas transcription factor E2 assists in the loading of E1 onto the viral origin, thus participating in the initiation of DNA replication (reviewed in [5]).

Due to their crucial functions, E1 and E2 proteins are in particular focus to intervene with HPV-related pathologies. Only a few chemical inhibitors targeting either ATP-ase activity of E1 proteins or E1/E2 interaction are available [6]. However, these inhibitors are either inefficient in cell-based assays or highly specific for particular HPV types. In addition, two small molecule inhibitors targeting cellular topoisomerase function have been shown to inhibit the replication of multiple HPV types [7]. On the other hand, E1 and E2 are phosphoproteins, and adjustment of their activities to support replication of the viral genome relies on recruitment of host cell protein kinases that might represent secondary targets to achieve inhibition of HPV replication. Targeting protein kinases has been proven to be a very effective way to modulate cellular physiology and thereby treat a number of disorders. Imanitib (BCR-Abl antagonist) and gefitinib (EGF receptor antagonist) have been identified as excellent examples of protein kinase inhibitor drugs (reviewed in [8]).

Protein kinases that have been shown or suspected to be involved in the regulation of E1 and/or E2 activities and cellular localization include protein kinases A and C, MAP kinases, casein kinases 1 and 2, cyclin dependent kinase (cdk) and FGF receptor 3 [9][10][11][12][13][14][15][16]. Casein kinase 2 (CK2) is an ubiquitously expressed dual specificity protein kinase involved in almost all aspects of cellular life. Additionally, CK2 is an anti-apoptotic kinase upregulated in multiple cancers (reviewed in [17]). It has been shown that CK2-dependent phosphorylation of bovine papillomavirus (BPV) E1 and E2 leads to loss of their DNA binding activity and attenuation of viral DNA replication [12]. Phosphorylation of putative CK2 sites in the hinge region of BPV E2 protein leads to its proteasomal degradation and reduction of BPV replication [18]. In contrast, CK2-mediated phosphorylation of the cellular protein BRD4 facilitates its interaction with HR HPV E2 proteins, which is required for proper segregation of the viral genome during mitosis, thereby rendering a positive effect on virus infection [19][20]. Although CK2-mediated inhibition of E1 DNA binding activity has been shown to be conserved *in vitro* in HPV11 and HPV31, the impact of CK2 on the replication of HPV genomes has remained obscure.

In cells, CK2 is mainly present as a heterotetrameric holoenzyme consisting of two catalytic (α and/or α´, are encoded by the *CSNK2A1* and *CSNK2A2* genes, respectively) and two regulatory (ββ, is encoded by the *CSNK2B* gene) subunits. Additionally, CK2 subunits may function independently of the holoenzyme (reviewed in [21]). Despite the overall similarity and overlapping biochemical characteristics, CK2α and CK2α´ isoforms exhibit a number of functional specializations, such as affinity to the β subunit, cellular localization, phenotypic differences of knock-out mice, involvement of CK2α´ in cellular proliferation, and specific interactions of the unique C-terminus of CK2α [22][23][24][25]. There is also evidence for differential substrate specificity of CK2 subunits: for instance, caspase-3 is phosphorylated exclusively by CK2α´ kinase and only in the absence of CK2β [26]. It is not yet clear whether this phenomenon is an exception to a rule, since most studies have explored the activity of only one CK2 protein, mainly CK2α, further extrapolating the obtained data to both catalytic subunits.

In the present study, we analyzed the impact of CK2 on the replication of different HPV genomes using *CK2* RNAi and CX4945-mediated inhibition of CK2 catalytic activity. We have taken advantage of the U2OS cells that efficiently support the replication of various HPV types and used adult pooled normal human epithelial keratinocytes (NHEKs) as well as HPV31b-positive CIN612 cells [27]. Our study reveals that efficient replication of various HPV subtypes requires the CK2α subunit, which regulates the stability and nuclear retention of endogenous E1 protein in a kinase activity-dependent manner.

## Results

### The CK2α catalytic subunit is required for transient replication of the HPV5, HPV11, HPV16, HPV18 and HPV31 genomes in U2OS cells

**Fig 1:**
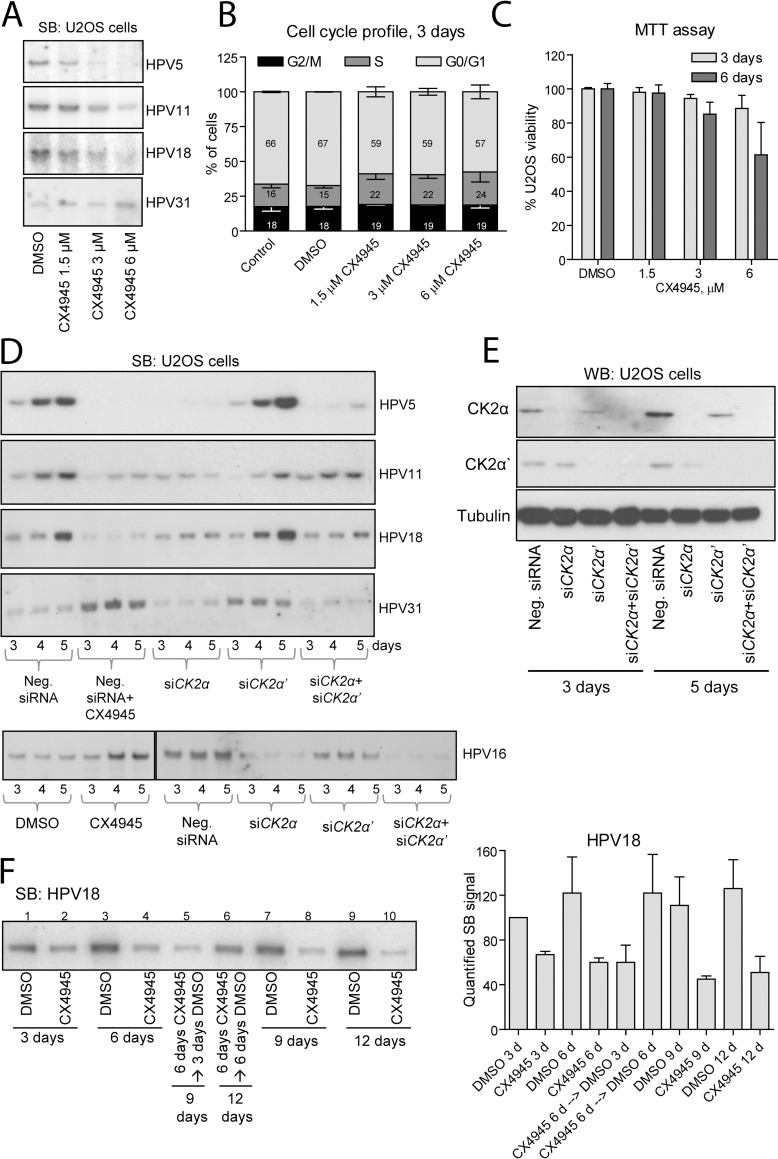
CK2α is required for transient replication of different HPV types. A. U2OS cells were transfected with HPV genomes and the next day treated either with vehicle or different concentrations of CX4945 for 48 h. Total DNA was extracted from cells, digested with DpnI and restriction enzymes to linearize the HPV genomes, and analyzed using Southern blot (SB). B, C. U2OS cells were transfected with HPV18 genome, next day treated with different concentrations of CX4945 and incubated for the indicated periods of time. Cell cycle profile was analyzed using propidium iodide by flow cytometry. Viability of the cells was examined using MTT assay. D. U2OS cells were transfected with different HPV genomes and siRNAs simultaneously using electroporation. Treatment with 6 μM CX4945 was started 24 h post transfection. Levels of the linearized replicated HPV genomes were analyzed using SB. E. U2OS cells were transfected with HPV18 and siRNAs specific for *CK2α* and *CK2α’* or scrambled Neg. siRNA simultaneously and incubated for 3 or 5 days. The cells incubated for 5 days, were transfected with the same siRNAs on the 3^rd^ day of incubation. Levels of CK2α, CK2α’ and tubulin proteins were detected using WB. F. left panel: U2OS cells were transfected with HPV18 genome, the next day treated with 6 μM CX4945, incubated for 3, 6, 9 and 12 days and analyzed using SB (lanes 1, 2, 3, 4, 7, 8, 9 and 10). One set of the cells treated with CX4945 for 6 days was switched to treatment with DMSO for additional 3 and 6 days that corresponded to 9 and 12 days post transfection (lanes 5 and 6). Right panel: SB signals from three independent experiments were quantified and set as 100% in the sample treated with DMSO for 3 days. Data are presented as an average mean +/- SD.

Several lines of evidence suggest the involvement of CK2 in the regulation of the PV life cycle. However, the direct impact of CK2 on the replication of different HPV types has not been analyzed. Additionally, it is not clear whether the catalytic activity of CK2 is involved in the regulation of the HPV life cycle. To analyze the overall impact of CK2 catalytic activity on HPV replication, we tested the HPV5 (β genus, cutaneous), HPV11 (α genus, LR, mucosal), HPV18 (α genus, HR, mucosal) and HPV31 (α genus, HR, mucosal) genomes in a transient replication assay in U2OS challenged with different concentrations of ATP-competitive small molecule CK2 inhibitor CX4945 (Fig 1A). Our data revealed that CX4945 suppresses the replication of the HPV5, HPV11 and HPV18 genomes in a concentration-dependent manner. In contrast, replication of HPV31 was not downregulated in the presence of CX4945. Level of HPV31 increased in response to 6 μM CX4945 suggesting that CK2 kinase activity may have a variable impact on different HPV types. Efficiency of CX4945 was tested by analyzing the status of CK2β subunit known to be phosphorylated by CK2 catalytic subunits [28] (S1A Fig). CK2β was over-expressed in U2OS cells treated with 3 and 6 μM CX4945 for 24 h. Whole cell extracts (WCEs) were analyzed using Western blot (WB). Two bands, with the phosphorylated form of CK2β migrated above the non-phosphorylated protein were detected in the control cells, whereas the level of the phosphorylated form of CK2β was severely reduced in the cells treated with 3 and 6 μM CX4945.

To rule out the possibility that inhibition of the HPV replication by CX4945 is a result of alterations in the cell cycle progression, we analyzed the cell cycle profiles of the HPV18-transfected U2OS cells incubated for 2, 3 or 6 days in the presence of dimethyl sulfoxide (DMSO) or different concentrations of CX4945 (S1B Fig, Fig 1B, and S1C Fig, respectively). During the course of the experiment, the cell cycle profile shifted towards G0/G1 phase that is a common feature of a cell culture with continuously increasing cell density (62%, 66% and 78% of the cells were detected in the G0/G1 phase at 2, 3 and 6 days, respectively). In contrast, CX4945 induced a slight shift of the cell cycle towards G2/M and S phases at the expense of cells in the G0/G1 phase. We propose that these small changes are not significant and therefore are not likely to be responsible for the changes in virus replication observed.

The viability of U2OS cells challenged with different concentrations of CX4945 was examined using methylthiazolyldiphenyl-tetrazolium bromide (MTT) assay (Fig 1C). Our results showed that the viability of U2OS cells remained similar to the control cells in all samples except the cells treated with the highest concentration of CX4945 (6 μM) for 6 days that resulted in approximately 40% loss of viable cells. Nevertheless, the negative effect of CX4945 on HPV replication is clearly detectable on the 3^rd^ day of incubation when the viability of the cells was similar to the control (Fig 1A and 1C). Additionally, in contrast to HPV5, HPV11 and HPV18, replication of HPV16 and HPV31 increased in response to 6 μM CX4945 during prolonged incubation (Fig 1A and 1D). Therefore, the reduced viability of the U2OS cells in the presence of 6 μM CX4945 could not lead to the inhibition of replication of the particular HPV types.

To test the involvement of CK2α and CK2α’ catalytic subunits in the regulation of HPV replication, we applied RNAi using siRNAs specific to either of the CK2 catalytic subunits. U2OS cells were transfected with HPV5, HPV11, HPV18, HPV31 and HPV16 genomes and *CK2α*- and/or *CK2α’*-specific siRNAs. Negative siRNA (Neg. siRNA) was used as a control. One set of cells transfected with Neg. siRNA was treated with 6 μM CX4945 to assess the impact of CK2 catalytic activity on HPV replication during prolonged incubation. Levels of replicated HPV genomes were analyzed using Southern blot (SB) after 3, 4 and 5 days of incubation (Fig 1D). The levels of all tested HPVs increased in time in the samples treated with Neg. siRNA confirming that HPV genomes replicate in the proliferating U2OS host cells and that their copy number per cell increases over time. Similar to our previous results (compare Fig 1A and 1D), CX4945 effectively inhibited the replication of the HPV5, HPV11 and HPV18, but upregulated the replication of the HPV31 and HPV16 genomes. Interestingly, silencing of the *CK2α* subunit led to strong inhibition of replication of all tested HPVs, including HPV31 and HPV16. A similar effect was observed in cells treated with a mix of si*CK2α* and si*CK2α’*. In contrast, knockdown of *CK2α’* had no negative effect on replication of all tested HPVs except HPV16. Nevertheless, *CK2α* RNAi had more profound negative effect even in the case of HPV16. To express the effects of *CK2* RNAi and CX4945 challenges in quantitative terms, all SB panels from at least 3 independent experiments obtained for each HPV type were quantified (S2 Fig). The obtained results confirmed the conclusions drawn. Taken together, our data indicate that only the CK2α subunit acts as a positive regulator of HPV5, HPV11, HPV16, HPV18 and HPV31 transient replication in U2OS cells, whereas CK2α’ is not required for replication of these HPV types.

The efficiency of si*CK2α*, si*CK2α’* and the mixture of the two was tested in U2OS cells using WB (Fig 1E). Levels of CK2 catalytic subunits remained below the detection limit in the samples treated with the respective siRNAs.

To test the long-term effect of CX4945 on the replication of the HPV18 genome and perform a rescue experiment, U2OS cells were transfected with HPV18 genome, the next day treated with CX4945 and incubated for 3, 6, 9 or 12 days. One set of the cells challenged with CX4945 for 6 days was switched to treatment with DMSO and incubated for additional 3 or 6 days (9 and 12 days post transfection in total, respectively). The level of HPV18 replication was assayed using SB (Fig 1F left panel), and SB signals from three independent experiments were quantified (Fig 1F right panel). After the 6^th^ day of incubation, HPV18 copy number remained constant (Fig 1F lanes 3, 7 and 9). The level of HPV18 was similar in all samples treated continuously with CX4945 (Fig 1F lanes 2, 4, 8, 10). However, termination of treatment with CX4945 led to increase of HPV18 copy number after 6 days of rescue (Fig 1F lane 6 vs 5 and 10).

### Overexpression of the CK2α subunit stimulates the replication of HPV11 and HPV18 in U2OS cells

**Fig 2:**
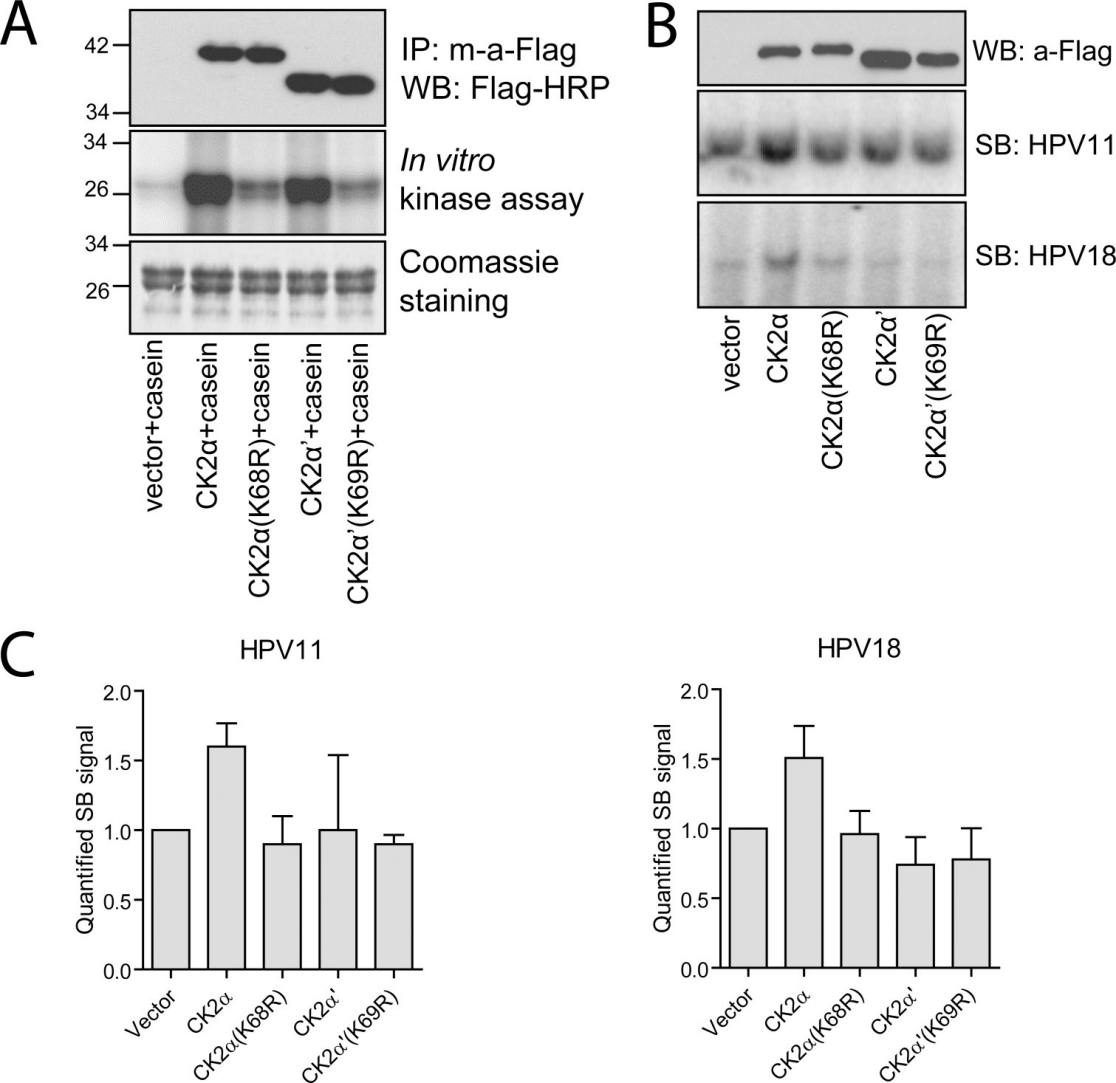
Overexpression of catalytically active CK2α enhances HPV11 and HPV18 replication. A. Flag-tagged CK2α, CK2α’ and their mutants were overexpressed in 293T cells, immuno-purified and subjected to WB analysis and *in vitro* kinase assay in the presence of casein. B. U2OS cells were co-transfected with either HPV11 or HPV18 genomes, Flag-tagged CK2α, CK2α’, their mutants or empty vector. Levels of CK2α, CK2α’ and their mutants were detected using WB and a-Flag antibody (upper panel). Total DNA was isolated 3 days after transfection, treated with DpnI and restriction enzymes to linearize HPV genomes and analyzed using SB. C. U2OS cells were treated as it is described in the panel B. The signals corresponding to the replicated HPV11 and HPV18 genomes were quantified and set as 1 in the samples transfected with an empty vector (left and right panels, respectively). Data are presented as the average mean +/- SD.

Depletion of the CK2α subunit in cells had a profound negative effect on HPV replication. Next, we performed an opposite experiment and analyzed the impact of CK2 overexpression on HPV replication. We generated constructs encoding N-terminally Flag-tagged wild-type (wt) CK2 subunits and their mutants CK2α(K68R) and CK2α’(K69R) bearing point mutations in their ATP binding pockets. Catalytic activity of the overexpressed and immuno-purified proteins was tested *in vitro* using casein as a substrate (Fig 2A). Flag-tagged CK2α and CK2α’ proteins were confirmed to be active kinases able to phosphorylate casein *in vitro*. In contrast, CK2α(K68R) and CK2α’(K69R) mutants demonstrated severely reduced catalytic activity. However, compared to an empty vector, some residual activity was observed suggesting that the indicated point mutation does not completely destroy kinase activity of CK2 subunits. Of the four CK2 proteins overexpressed, only wt CK2α was able to enhance HPV11 and HPV18 replication in U2OS cells (~1.5–1.6 times) (Fig 2B and 2C). These results are consistent with the results obtained in RNAi and CX4945 inhibitor experiments, and indicate that it is the presence and activity of CK2α that modulate HPV11 and HPV18 DNA replication.

### Generation and testing the HPV18NLuc marker genome as a tool for screening HPV replication

Replication of HPV genomes is generally analyzed using either SB or qPCR. These time consuming and expensive methods require purification of total or extrachromosomal DNA prior analysis. Recently, luciferase HPV DNA replication readout assays have been developed [29]; and an HPV18RLuc construct was generated and used for screening for small molecule inhibitors of the HPV life cycle [7]. To make the test system more sensitive, we generated a novel HPV18 genome containing NanoLuc (NLuc) encoding sequence in frame with E2 introduced immediately after the E1 stop codon, followed by foot-and-mouth disease virus (FMDV) 2A sequence and full-length E2 ORF. The resulting HPV18NLuc construct expresses NLuc fused to the first 24 E2 amino acids N-terminally and self-processed FMDV-2A peptide sequence C-terminally. The inserted NLuc/FMDV-2A encoding sequence enlarges the genome size only by 657 bp, which is much smaller, as compared to the previously reported HPV18RLuc genome (the inserted sequence was 1077 nt). Therefore, this construct can also be a valuable tool to test HPV cell entry inhibitors in a high-throughput format. Additionally, HPV5NLuc and HPV11NLuc constructs were generated using the same approach. The maps of the HPV5NLuc, HPV11NLuc and HPV18NLuc constructs are depicted in S3A Fig.

**Fig 3:**
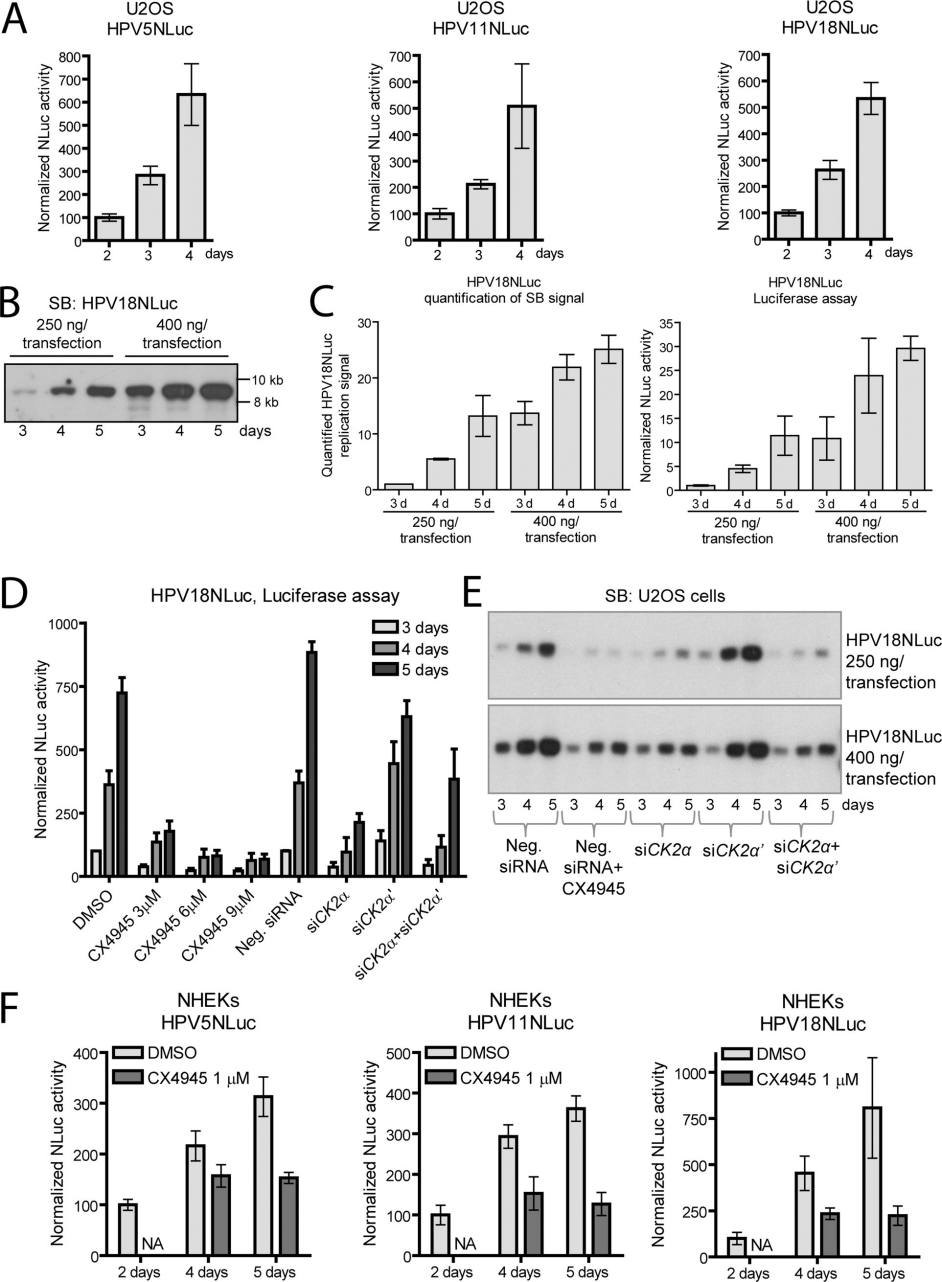
Replication of the HPV18NLuc genome is regulated by CK2α. A. U2OS cells were transfected with different HPVNLuc genomes, propagated for 2, 3 and 4 days and subjected to luciferase assay. Nluc activity was normalized to alkaline phosphatase activity. Normalized Nluc activity was set as 100% in the cells propagated for 2 days. B. U2OS cells were transfected with 250 or 400 ng of the HPV18NLuc genome and incubated for 3, 4 and 5 days. Total DNA was isolated and treated with DpnI and BglI. Replication of HPV18NLuc genome was analyzed using SB. C. U2OS cells were transfected with different amounts of HPV18NLuc genome and FFLuc encoding plasmid and incubated for the indicated periods of time. Signals of HPV18NLuc replication were quantified using ImageQuant software; the average means of three experiments +/- SD are shown (left panel). The NLuc activity was measured and normalized to FFLuc activity (right panel). Signals of HPV18NLuc replication or normalized NLuc activity were set as 1 in the sample transfected with 250 ng of HPV18NLuc and incubated for 3 days. D. U2OS cells were transfected with HPV18NLuc genome, FFLuc encoding plasmid and siRNAs if indicated. The next day, cells were treated with different concentrations of CX4945 or vehicle. Cells were incubated for 3, 4 or 5 days and subjected to luciferase assay. NLuc activity was normalized either to alkaline phosphatase activity in the samples treated with CX4945 or to FFLuc and alkaline phosphatase activities in the samples treated with siRNAs. Normalized NLuc activity in the cells treated with DMSO and incubated for 3 days was set as 1. E. U2OS cells were transfected with different amounts of HPV18NLuc genome and siRNAs. The next day, the cells were treated with 6 μM CX4945 or vehicle. Total DNA was extracted, treated with DpnI and BglI and analyzed using SB. F. NHEKs were transfected with different HPVNLuc genomes, incubated for 48 h and treated with 1 μM CX4945, if indicated. Nluc activity was normalized to total protein concentrations and set as 100% in the samples incubated for 2 days after transfection. NA–not analyzed. All panels: normalized NLuc data are presented as an average mean +/- SD of at least 3 replicates measured in 3 independent experiments.

Replication of HPV5NLuc, HPV11NLuc and HPV18NLuc was tested in U2OS cells using luciferase assay (Fig 3A). All of these genomes replicated in U2OS cells as were assessed by increase of normalized NLuc activity at 2, 3 and 4 days after transfection.

We analyzed the behavior of HPV18NLuc genome in more details. First, replication efficiency of HPV18NLuc construct was compared to that of the wt HPV18 genome in U2OS cells. Our data revealed that the mutant and wt genomes replicated similarly (S3B Fig). Next, we transfected U2OS cells with 250 or 400 ng of HPV18NLuc genome in combination with firefly luciferase (FFLuc) encoding plasmid and analyzed the replication efficiency of the HPV18NLuc using SB after 3, 4 and 5 days of incubation (Fig 3B). The data from these experiments confirmed that the HPV18NLuc genome replicated similarly to the wt HPV18 genome (Figs 3B and 1D, respectively). SB signals corresponding to the replicated HPV18NLuc genome were quantified using ImageQuant software (Fig 3C left panel). Alternatively, cells were seeded on a 96-well plate, and NLuc and FFLuc activities were measured at the respective time points (Fig 3B right panel). In both cases, the signals obtained in the cells transfected with 250 ng of the HPV18NLuc genome and incubated for 3 days were set as 1. Normalized NLuc activity correlated well with quantified SB signals. Linear regression analysis revealed a statistically significant correlation (R^2^ = 0.96, p<0.0005) (S3C Fig) indicating that normalized NLuc activity may be used for the analysis of HPV18NLuc genome replication.

Replication of the HPV18NLuc genome was inhibited by CX4945 and *CK2* RNAi in both luciferase and SB assays (Fig 3D and 3E, respectively). We tested 3 different concentrations of CX4945: 3, 6 and 9 μM. The luciferase assay showed that 6 and 9 μM CX4945 had similar effects, therefore usage of concentrations higher than 6 μM was not necessary. Replication of HPV18NLuc in cells lacking CK2α was also severely reduced regardless of the initial amount of the genome used for transfection (Fig 3E). Efficiency of the *CK2* siRNAs in U2OS cells harboring the HPV18NLuc genome was controlled using WB (S3D Fig).

The obtained results were confirmed using HPV5NLuc, HPV11NLuc and HPV18NLuc in the NHEKs (Fig 3F). The cells were transfected with the respective genomes, incubated for 2 days, treated with 1 μM CX4945 or vehicle for additional 2 or 3 days and subjected to the luciferase assay. All tested genomes replicated in NHEKs, and CX4945 effectively inhibited their replication. Taken together, our data introduce a novel fast, sensitive and reliable test system suitable for studying HPV replication in quantitative terms in both U2OS cell line and normal human keratinocytes. Our results clearly show that CX4945 inhibits replication of these genomes, although in principle addition of NLuc sequence into HPV genomes might also affect viral transcription, splicing, mRNA stability and translation.

### Stable replication of the HPV31b genome depends on CK2α in CIN612 cells

**Fig 4:**
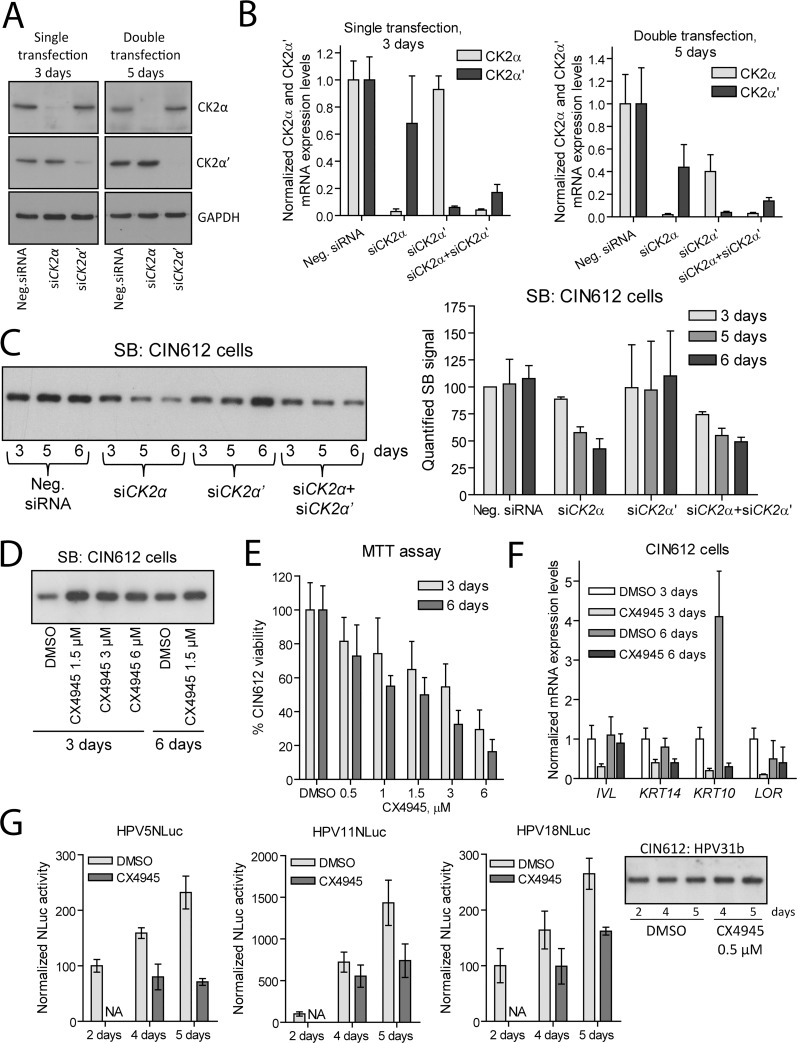
CK2α regulates replication of the HPV31b genome in CIN612 cells. A. CIN612 cells were transfected with siRNAs one or two times and propagated for 3 or 5 days. Levels of CK2α and CK2α’ proteins were detected using WB. B. CIN612 cells were transfected with siRNAs as is described in the panel A. Levels of *CK2α* and *CK2α’* mRNA expression were measured using qPCR, normalized to *GAPDH* expression level and set as 100% in a sample transfected with Neg. siRNA. Data from other samples were calculated relative to that. Data are presented as the average mean +/- SD of three independent experiments performed in triplicate. C. CIN612 cells were subjected to single or double transfection with siRNAs and incubated for 3, 5 and 6 days, respectively. Total DNA was extracted, treated with HindIII restriction enzyme and subjected to SB analysis (left panel). SB signals obtained from three independent experiments were quantified, and data from the sample treated with Neg. siRNA and incubated for 3 days was set as 100% (right panel). D. CIN612 cells were treated with the indicated concentrations of CX4945 for 3 or 6 days. Total DNA was digested with HindIII restriction enzyme to linearize the HPV31b genome and subjected to SB analysis. E. Viability of CIN612 cells incubated in the presence of different concentrations of CX4945 for 3 or 6 days was tested using MTT assay. F. CIN612 cells were treated with 0.5 μM CX4945 for 3 or 6 days. The levels of the respective gene mRNA expression were measured by qPCR using 2 different pairs of primers, normalized with *GAPDH* mRNA expression levels and set as 1 in the samples treated with DMSO for 3 days. G. CIN612 cells were transfected with different HPVNLuc genomes, incubated for 2 days and treated with 0.5 0.5 μM CX4945 for additional 2 and 3 days. NLuc activity was measured, normalized to total protein concentrations and set as 100% in the samples incubated for 2 days. NA–not analyzed. The linearized HPV31b genome was analyzed using SB in the respectively treated samples (right panel). All panels: data are presented as an average mean +/- SD.

To confirm the obtained results in another cell culture model system supporting HPV replication, we chose HPV31b+ CIN612 keratinocytes. First, we treated CIN612 cells with *CK2*-specific siRNAs one or two times and tested CK2α and CK2α’ protein levels using immunoblotting (Fig 4A). After the first transfection, both proteins were fairly detected, whereas the double transfection resulted in complete loss of expression of both proteins indicating that *CK2* RNAi is functional in CIN612 cells. To be convinced that both antibodies recognize the indicated CK2 proteins, we also monitored the levels of *CK2α* and *CK2α’* mRNAs (Fig 4B) in CIN612 cells subjected to single or double transfection with *CK2* siRNAs (Fig 4B left and right panels, respectively). Levels of *CK2α* and *CK2α’* mRNAs were reduced more than 90 and 95% after the 1^st^ and 2^nd^ transfection, respectively.

Next, CIN612 cells were transfected with *CK2*-specific siRNAs; total DNA from the transfected cells was treated with the restriction endonuclease linearizing the HPV31b genome, and subjected to SB analysis (Fig 4C left panel). *CK2α* RNAi induced clear downregulation of HPV31b stable replication in CIN612 keratinocytes, similar to the HPV31 transient replication in U2OS cells. These data were confirmed by quantification of the SB signal obtained from 3 independent experiments (Fig 4C right panel).

To test the effect of CX4945 on stable replication of HPV31b, CIN612 cells were treated with different concentrations of CX4945, incubated for 3 or 6 days and subjected to total DNA isolation and SB analysis (Fig 4D). We could not detect any CX4945-mediated negative effect on HPV31b replication (Fig 4D). Moreover, similarly to transient replication of HPV31 in U2OS cells (Fig 1A and 1D), we observed some upregulation of HPV31b copy number in the presence of CX4945. However, even the lowest concentration of CX4945 used (1.5 μM) was toxic to the cells, as was assessed by MTT assay (Fig 4E). Therefore, we could not analyze the effects of 3 or 6 μM CX4945 on replication of HPV31b during prolonged incubation.

One likely explanation for the failure of CX4945 to suppress HPV31b replication in CIN612 keratinocytes relies on its putative ability to induce differentiation of these cells, although it does not explain the CX4945-mediated positive effect on transient replication of HPV31 in U2OS cells. This ability could also explain the slight increase in the HPV31b copy number in CX4945-treated cells, since differentiation of CIN612 cells results in an increase in viral genomes per cell [30]. Support for this hypothesis comes from the study showing that a CK2 inhibitor similar to CX4945 induces differentiation of normal human keratinocytes at least partially in a CK2-independent manner [31]. To rule out this possibility, we analyzed the keratinocyte-specific gene mRNA expression levels in CIN612 cells treated either with DMSO and 0.5 μM CX4945 or transfected with Neg. siRNA and si*CK2α* once or twice and incubated for 3 and 6 days, respectively (Fig 4F and S4A Fig, respectively). Expression levels of *keratin 10 (KRT10)*, *keratin 14 (KRT14)*, *involucrin (IVL)* and *loricrin (LOR)* mRNAs were analyzed. Neither CX4945 nor *CK2α* RNAi could induce expression of these genes indicating that differentiation of CIN612 cells was not induced by these treatments.

Also, we analyzed the cell cycle profile in the respectively treated CIN612 cells (S4B Fig). Cell cycle profiles were similar in the cells treated with CX4945 and DMSO for 3 days. Similar to the results obtained using U2OS cells, CX4945 induced a shift towards G2/M phase in the case of prolonged incubation (Fig 1B and S4B Fig). However, a number of cells in the S-phase did not change substantially in time in contrast to the control DMSO-treated cells, which demonstrated strong increase of the cells in the S-phase (approximately 2.4 times). Compared to the cells transfected with Neg. siRNA, knockdown of CK2α led to a decrease in cell number in the S-phase (approximately 40 and 45% after 3 and 6 days of incubation, respectively). Therefore, we cannot formally rule out the possible association between the changes in the cell cycle progression and inhibition of HPV31b replication induced by *CK2α* RNAi. Nevertheless, all our SB analyses were normalized to the amount of the genomic DNA, thereby indicating that we are measuring the HPV31b copy number per cell.

Finally, we were interested to examine the ability of CX4945 to inhibit the replication of other HPV types in CIN612 cells. CIN612 cells were transfected with HPV5NLuc, HPV11NLuc and HPV18NLuc genomes, incubated for 2 days, treated with 0.5 μM CX4945 for additional 2 and 3 days and subjected to luciferase assay (Fig 4G left panels). Also, total DNA was isolated and subjected to SB analysis to detect HPV31b replication at the same conditions (Fig 4G right panel). As it was expected, replication of HPV31b was not suppressed by 0.5 μM CX4945 in time. However, similar to the results obtained in NHEKs and U2OS cells, CX4945 inhibited the replication of HPV5NLuc, HPV11NLuc and HPV18NLuc in CIN612 cells.

### CX4945 and *CK2α* RNAi induce degradation of HPV11 and HPV18 E1 helicase

**Fig 5:**
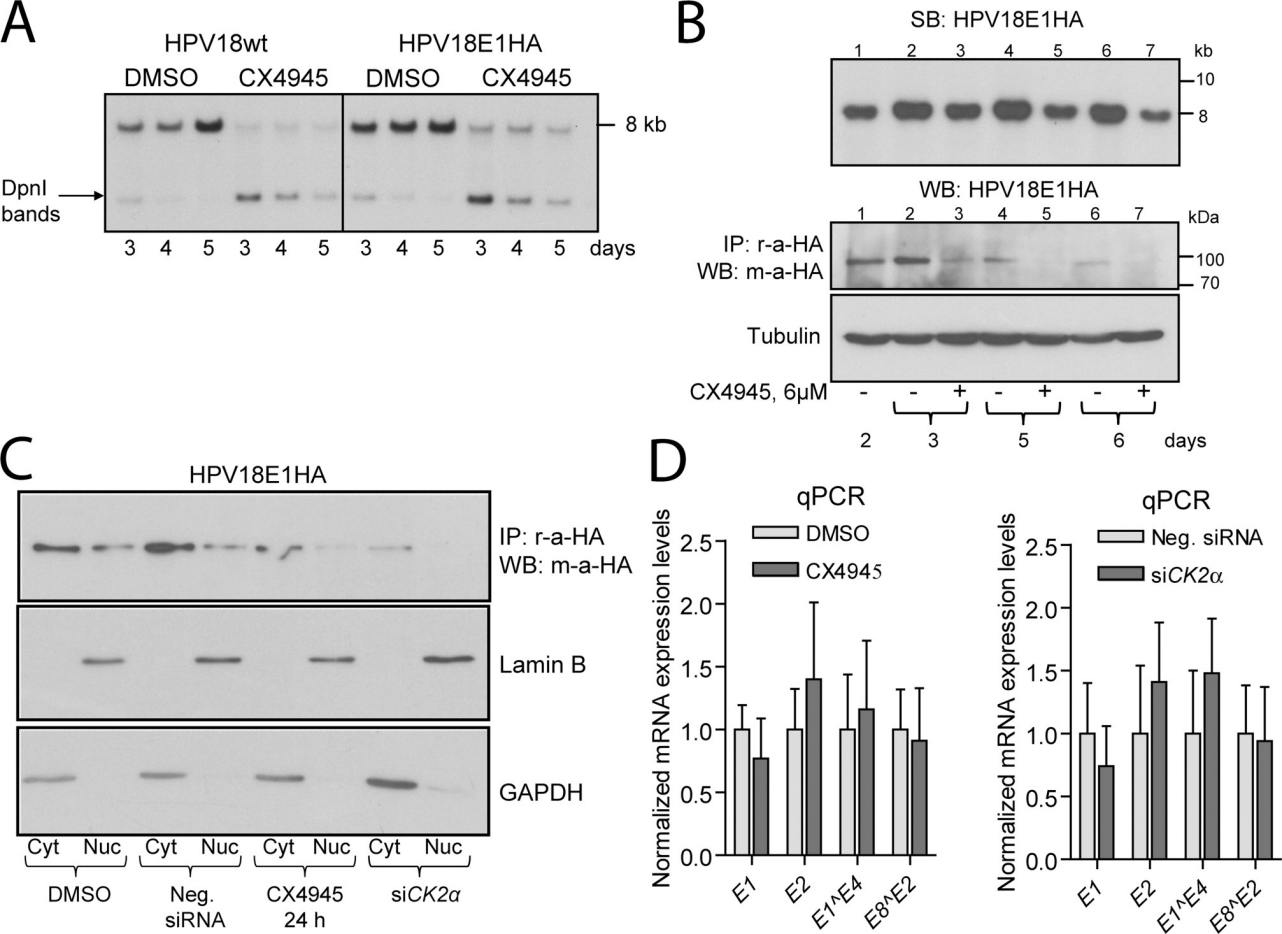
CK2α catalytic activity is required for stability of the HPV18 E1 protein. A. U2OS cells were transfected with HPV18E1HA and HPV18wt genomes, the next day treated with 6 μM CX4945 or vehicle and propagated for 3, 4 and 5 days. Total DNA was extracted, digested with DpnI and BglI restriction enzymes and analyzed using SB. Low molecular weight bands correspond to DpnI-sensitive input DNA. B. U2OS cells were transfected with the HPV18E1HA genome and propagated for 2, 3, 5 and 6 days. CX4945 was added to cells 2 days after transfection. Cells were fractionated for isolation of total DNA and WCEs. Total DNA was treated with DpnI and BglI restriction enzymes and analyzed using SB. HA-tagged E1 protein was immunoprecipitated from WCEs and analyzed using WB. Tubulin was used as a loading control. C. U2OS cells were transfected with HPV18E1HA construct and siRNAs, if indicated, incubated for 3 days and treated with 6 μM CX4945 for 24 h. Cells were detached using trypsin-EDTA and fractionated for nuclear (Nuc) and cytoplasmic (Cyt) extracts. Nuclear and cytoplasmic HA-tagged E1 protein was immunoprecipitated using r-a-HA antibody and analyzed by immunoblotting using m-a-HA antibody. The nuclear and cytoplasmic markers lamin B and GAPDH were used. D. U2OS cells were transfected with the HPV18 genome, incubated for 2 days and treated with 6 μM CX4945 for 24 h (left panel). Alternatively, U2OS cells were cotransfected with the HPV18 genome and indicated siRNAs (right panel). Total RNA was extracted, treated with Turbo DNase and used for cDNA synthesis. Levels of *E1*, *E2*, *E1^E4* and *E8^E2* transcripts were measured using qPCR, normalized to *GAPDH* expression level and set as 100% in samples treated with DMSO or transfected with Neg. siRNA. Data are presented as the average means +/- SD of three independent experiments performed in triplicate.

Helicase E1 and transcription factor E2 are the only viral proteins required for PV replication. It has been shown that CK2 is able to directly phosphorylate the BPV1 E1 and E2 protein, modulating their activities [12]. Since our data show that CK2α is required for efficient replication of different HPV types, we hypothesized that CK2α may mediate its effects via E1 and/or E2 proteins. Transient replication assays of HPV11 and HPV18 revealed that CX4945 mimics the effect of si*CK2α* and may be used as an advantageous alternative to *CK2α* RNAi. First, pharmacological intervention with CK2 kinase activity influences all cells in the population. Second, it is much faster than RNAi-mediated knockdown of protein expression, which effectiveness depends on transfection efficiency. To gain insight into the molecular mechanisms of CK2-dependent downregulation of HPV replication, we generated HPV11 and HPV18 genomes containing HA epitope encoding sequence in their E1 ORFs after the 15^th^ nucleotide (HPV11E1HA and HPV18E1HA, respectively). Since the HA epitope could potentially influence the properties of the E1 protein, the replication of these constructs was compared with that of the respective wt genomes in U2OS cells challenged with 6 μM CX4945 or vehicle (Fig 5A and S5A Fig). Our data revealed that the mutant and wt genomes replicated similarly in both cases indicating that the functioning of wt and HA-tagged E1 is similar.

Next, we analyzed the level of HA-tagged E1 protein in U2OS cells treated with 6 μM CX4945 on the 2^nd^ day after transfection and incubated for the additional 1, 3 or 4 days. Concurrently, the level of linearized HPV DNA was analyzed in the same samples using SB. Data from the representative experiments are shown in Fig 5B and S5B Fig for HPV18E1HA and HPV11E1HA, respectively. In contrast to CX4945-treated cells, the levels of HPV18E1HA and HPV11E1HA DNA increased in time in the control cells. We failed to detect the HA-tagged E1 proteins by WB directly from WCEs, but immunoprecipitated E1 protein was detected as a single band migrating at approximately 90 kDa. The levels of E1 protein immunoprecipitated from cells challenged with CX4945 were reduced already after 24 h of treatment, whereas decrease in HPV DNA copy number was not detected at this time point (compare lanes 1 and 3 in SB and WB panels of Fig 5B and S5B Fig). Even further, HPV18 and HPV11 genomes were readily detectable after 72 h of treatment with CX4945 on day 5, whereas we were unable to detect any E1 protein at this time point (lanes 5 in SB and WB panels of Fig 5B and S5B Fig). These data suggest that the CK2 inhibitor induces the degradation of E1 protein.

Next, we analyzed the levels of nuclear and cytoplasmic HPV18 E1 protein in U2OS cells either transfected with si*CK2α* or treated with 6 μM CX4945 for 24 h. The nuclear and cytoplasmic extracts were isolated; the E1 protein of HPV18E1HA was immunoprecipitated and analyzed using WB (Fig 5C). Compared to the respective control cells, the levels of nuclear and cytoplasmic E1 protein in the cells treated with si*CK2α* or CX4945 were lower.

To exclude the possibility of transcriptional downregulation of *E1* expression, we analyzed the levels of HPV18-derived transcripts in U2OS cells either challenged with CX4945 for 24 h or transfected with si*CK2α*. E1 is encoded by the longest viral pre-mRNA that also includes ORFs of several other genes generated via alternative splicing [32]. The levels of mRNAs corresponding to HPV18 *E1*, *E2*, *E1^E4* and *E8^E2* transcripts were analyzed using RT-PCR and qPCR 48 h after transfection (Figs 5D and S5C, respectively). Our data showed that neither CX4945 nor si*CK2α* inhibited the transcription of the analyzed HPV18 genes.

**Fig 6:**
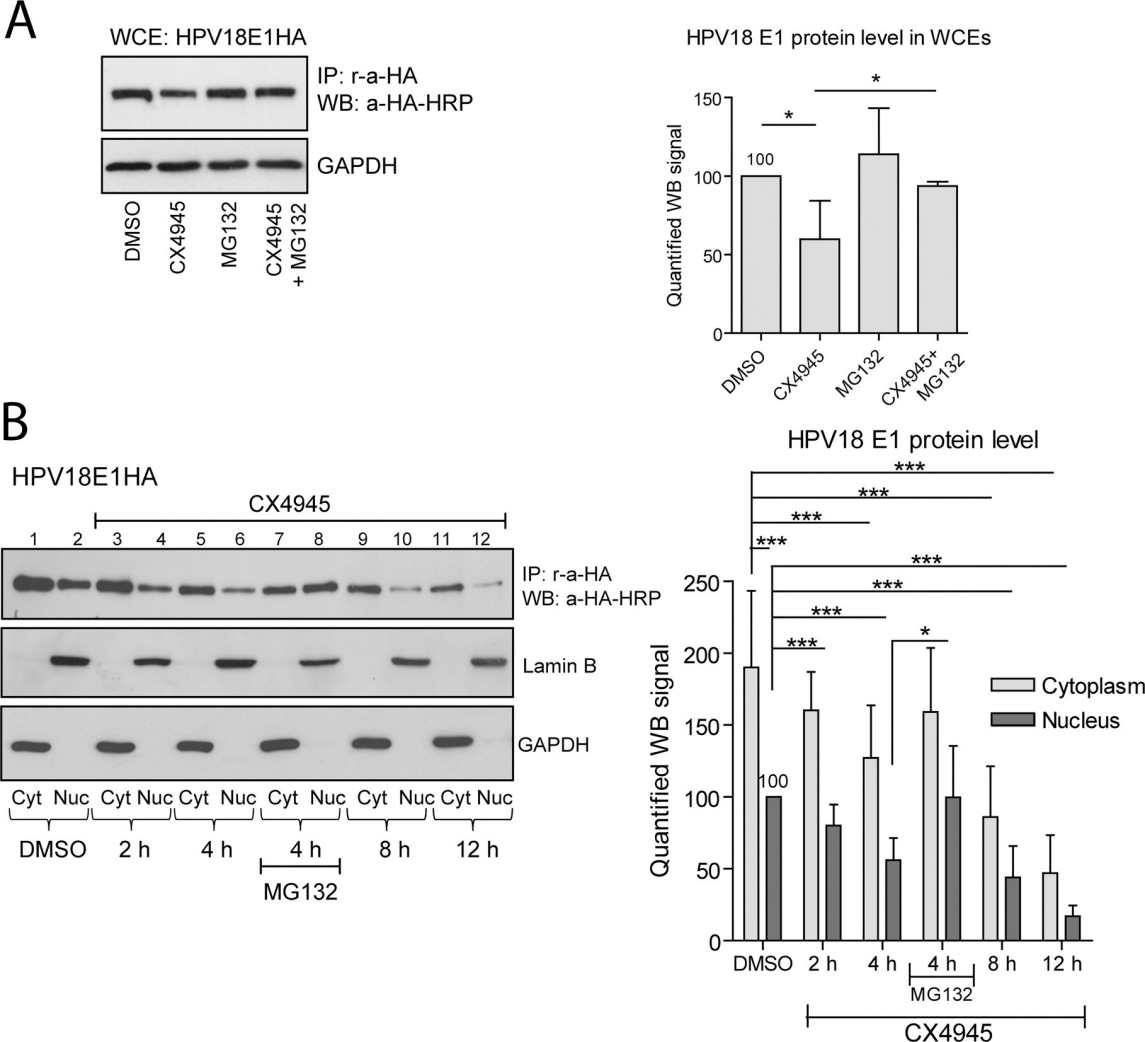
CX4945 induces proteasomal degradation of E1 protein. A. U2OS cells were transfected with the HPV18E1HA genome and incubated for 3 days. Cells were treated with 6 μM CX4945 and/or 10 μM MG132 for 6 h. HA-tagged E1 protein was immunoprecipitated from whole cell extracts (WCEs) using r-a-HA antibody and analyzed by immunoblotting using m-a-HA-HRP antibody (left panel). WB signals from three independent experiments were quantified. E1 level was set as 100% in the cells treated with DMSO (right panel). B. U2OS cells were transfected with the HPV18E1HA genome, incubated for 3 days and treated with 6 μM CX4945 (lanes 3–12) and 10 μM MG132 (lanes 7 and 8) for the indicated periods of time. Nuclear (Nuc) and cytoplasmic (Cyt) extracts were isolated. Nuclear and cytoplasmic HA-tagged E1 protein was immunoprecipitated using r-a-HA antibody and analyzed by immunoblotting using a-HA-HRP antibody. The nuclear and cytoplasmic markers lamin B and GAPDH were used (left panel). WB signals obtained from at least three independent experiments were quantified. The E1 protein level was set as 100% in the NE of cells treated with DMSO. All panels: data are presented as the average means +/- SD of at least three independent experiments (*—p<0.05; ***—p<0.001).

To confirm our observations and exclude the possibility of a CK2 independent “side” effect of CX4945 or a simple decrease in E1 levels in cells possessing fewer copies of HPV18E1HA or HPV11E1HA genomes, we analyzed the levels of the HA-tagged E1 protein of HPV18 or HPV11 in U2OS cells treated with CX4945 and proteasome inhibitor MG132. The HA-tagged E1 protein was immunoprecipitated and analyzed using immunoblotting and two different antibodies–m-a-HA (clone HA-7) or rat-a-HA-HRP (clone 3F10) (Fig 6A and S5D Fig for HPV18E1HA and HPV11E1HA, respectively). CX4945 induced decrease in E1 protein level was rescued to some extent in the presence of the proteasome inhibitor MG132, suggesting that E1 is subjected to proteasomal degradation in response to CK2 inhibitor. Also, the WB signals corresponding to HPV18 E1 protein were quantified and set as 100% in the cells treated with DMSO. The data from other samples were calculated relative to 100%. Statistical analysis was performed using T-test assuming equal variations (Excel), and two-tail p values were calculated (Fig 6A right panel). MG132 alone had no significant effect on HPV18 E1 protein levels. However, level of the E1 protein decreased approximately 50% in response to treatment with CX4945 for 6 h, which was rescued in the presence of MG132.

To study this phenomenon in further detail, we isolated the nuclear and cytoplasmic extracts of U2OS cells transfected with HPV18E1HA or HPV11E1HA genomes and treated with 6 μM CX4945 for 2, 4, 8 and 12 h. Along with CX4945 treatment, the cells transfected with the HPV18E1HA genome were treated with MG132 for 4 h. E1 proteins of HPV18E1HA and HPV11E1HA were immunoprecipitated and analyzed using immunoblotting (Fig 6B and S5D Fig, respectively). WB data from at least three independent experiments performed using HPV18E1HA genome were quantified. E1 level was set as 100% in the nuclear extracts of cells treated with DMSO. The analysis revealed significantly lower E1 levels in nuclear and cytoplasmic extracts within 4 h of treatment with CX4945 (Fig 6B right panel). The decrease of HPV18 E1 protein level was rescued by MG132 to the levels similar to the ones observed in the control cells. Although we could not fully exclude the possibility of N-terminal proteolysis of full-length E1 proteins, our data suggest that CK2α catalytic activity is required for posttranslational stabilization and nuclear retention of E1 proteins. In contrast to the E1 protein, the levels of nuclear and cytoplasmic CK2α and CK2α’ proteins were not markedly reduced in response to CX4945 in the HPV11E1HA transfected U2OS cells (S5D Fig).

## Discussion

Constitutively active protein kinase CK2 is generally considered to be a lateral signal modulator, rather than an integral part of most signal transduction pathways. CK2 is frequently deregulated and often overexpressed in cancer cells (reviewed in [33]). Despite the fact that hundreds of target sites have been identified for CK2, cells lacking both CK2 catalytic subunits are viable (reviewed in [17]). This property makes it a potential drug target for a number of diseases including cancer. Indeed, two inhibitors of CK2 (cyclic peptide CIGB-300 and ATP-competitive inhibitor CX4945) have reached clinical trials for the treatment of cancer [33].

In the present paper, we have analyzed the function of CK2 in regulating HPV replication. We show that CK2 activity is required for efficient transient and stable replication of a number of HPV types. We further show that only one of the catalytic subunits of the kinase, CK2α, is required for HPV replication. Finally, we show that the inhibition of CK2 activity induces the degradation of the E1 protein, a key replication factor of HPV.

### The CK2 inhibitor CX4945 efficiently suppresses the replication of a number of HPV types

First, we show that pharmacological inhibition of CK2 catalytic activity using the ATP-competitive inhibitor CX4945 leads to suppression of transient replication of HPV5, HPV11 and HPV18 in U2OS cells. Also, transient replication of HPV5NLuc, HPV11NLuc and HPV18NLuc genomes was inhibited by CX4945 in NHEKs and CIN612 cells. Nevertheless, CX4945 does not inhibit the transient replication of HPV16 and HPV31 genomes in U2OS cells as well as the stable replication of the episomal HPV31b genome in CIN612 cells. Instead, the copy number of HPV16, HPV31 and HPV31b genomes increased in response to CX4945. The fact that the replication of these genomes is not suppressed in response to CX4945 is puzzling. Several reasons might explain this phenomenon. First, although CX4945 is currently the best inhibitor for CK2 (IC_50_ 1 nM in kinase assay *in vitro*), it still targets a number of other kinases at nanomolar range [34]. It may be possible that an “off-target” kinase has a positive effect on HPV16 and HPV31 replication. Second, it has been shown that CX4945 has CK2 independent function as an inhibitor of alternative splicing [35]. Because the splicing patterns and translation efficiency of different polycistronic transcripts varies between HPV types, it is plausible to speculate that CX4945 may alter the translational outcome in a HPV-type-dependent manner. Alternatively, CK2 might possess additional, kinase activity-independent positive roles in the replication of a subset of HPV types, as exemplified by HPV16 and HPV31 in this paper. Such regulatory CK2 activity has been shown in melanoma cells, where CK2 acts as a scaffold to keep ERK kinase active [36]. The last hypothesis is supported by the fact that *CK2α* RNAi leading to loss of CK2α protein results in inhibition of HPV16 and HPV31 replication in U2OS and HPV31b replication in CIN612 cells. Further studies are needed to comprehensively explain the positive effect of CX4945 on HPV16, HPV31 and HPV31b replication.

The effects of small molecule modulators are often pleiotropic and might be indirect. It might be envisaged that CX4945 induces cell cycle alterations or loss of cell viability in U2OS cells and the effect on HPV replication is non-specific. We ruled out this possibility, by showing that the minor changes in cell cycle and viability of U2OS cells detected in the presence of CX4945 at the concentrations used could not be a reason of such dramatic inhibition of HPV5, HPV11 and HPV18 replication. Besides, replication of HPV16 and HPV31 was not inhibited at the same experimental conditions. Furthermore, the signals of our replication assays are normalized against the amount of genomic DNA, and therefore, the signal represents copies of the HPV genome per cell.

### CK2α, but not CK2α’, is a positive regulator of HPV replication

To obtain more precise insight into the function of CK2 in HPV replication, we used siRNA-mediated knockdown of both CK2 catalytic subunits, CK2α and CK2α’. Our results show that surprisingly only CK2α is required for efficient transient and stable replication of HPV genomes, although both subunits are present in the cells analyzed. Inactivation of CK2α’ had no or little effect on the replication efficiency of the tested HPV types. This result was corroborated by performing the opposite experiment and showing that overexpression of CK2α, and not CK2α’, stimulates transient replication of HPV11 and HPV18 genomes in U2OS cells. Additionally, we show that the catalytic activity of CK2α is required for this stimulation, since the mutant with impaired kinase activity was not able to enhance HPV replication.

CK2 generally functions as a heterotetrameric enzyme, consisting of two catalytic subunits (CK2α or CK2α’) and two regulatory CK2β subunits. Catalytic subunits are highly homologous (almost 90%) in their kinase domain and more divergent in C-termini. The identified consensus phosphorylation site is shared by both catalytic subunits (reviewed in [17]). Despite this fact, many interactors have been identified as specific to either of the CK2 catalytic subunits, indicating certain functional divergence [37]. Additionally, a number of substrates are phosphorylated by one or another of the subunits ([26] and references therein), although the sequence specificity underlying this phenomenon has not been discovered. In addition, some CK2 substrates are phosphorylated by the catalytic subunit only and not by the holoenzyme [38][26].

Our results showing that CK2α, and not CK2α’, regulates HPV replication leave two possible explanations. First, it is conceivable that one or more proteins participating in HPV replication harbor phosphorylation sites unique to CK2α. Second, it is possible that the levels of different CK2 subunits in the cells analyzed are very strongly in favor of CK2α’, and enough catalytically active CK2α’ remains in the cells even after siRNA-mediated knockdown of CK2α’ subunit. We believe the first possibility to be correct, since the residual expression of CK2α and CK2α’ mRNAs was similar (approximately 3–5%), and the levels of both proteins remained under their detection limits as assessed by immunoblotting in our RNAi experiments. Furthermore, we do not observe a cooperative negative effect on HPV replication if knock-down of both subunits is performed.

A positive role of CK2 in HPV replication has been observed previously [19]. This report shows that CK2-mediated phosphorylation of the cellular protein BRD4 is required for efficient replication of E1- and E2-dependent replication of the origin-containing plasmid in C33A cells. The role of phosphorylated BRD4 in replication most likely relies on correct targeting of the E2 protein to the origin of replication [20]. Our present paper confirms the requirement of CK2 in HPV replication and proves that this also holds true in the HPV genome context. Furthermore, our data show that in addition to the role of CK2 in transient replication, it also regulates stable replication of the HPV31b genome.

Another phenomenon we observed in the RNAi experiments was that inhibition of stable HPV31b replication in CIN612 cells was milder than inhibition of transient HPV31 replication in U2OS cells. This is expected since the HPV genome is duplicated in synchrony with cellular DNA during stable replication, whereas viral genome copy number is increasing during every cell cycle during initial transient replication. Therefore, the effect on stable replication should be smaller as the number of HPV genome replication initiation events is much less in a given period of time.

### HPV11 and HPV18 E1 proteins are degraded in response to CX4945 challenge

A number of studies have shown that BPV1 E1 and E2 proteins are phosphorylated by CK2 [39][12][13]. These studies were performed using recombinant viral proteins purified from *E*. *coli* or Sf9 cells followed by an *in vitro* kinase assay. Most of these identified sites are conserved in HPV E1 and E2 proteins. It has been shown that the consequence of these phosphorylations is the inactivation of DNA binding of both E1 and E2 [12]. In addition, BPV1 E1 is phosphorylated in putative CK2 sites *in vivo* while overexpressed in Sf9 cells, although it cannot be completely ruled out that phosphorylation takes place during purification steps and not in cells [13][40]. The *in vivo* function of these CK2 sites in BPV1 E1 and E2 proteins has been studied by mutating the sites in the context of BPV genomes and analyzing the consequences in C127 cells [12] [41]. The conclusions drawn from these experiments, however, differ between laboratories. One group has shown that alanine substitutions in putative CK2 sites in E1 have no effect on transient or stable replication of BPV, while mutations in E2 phosphosites have a positive effect on BPV replication [12]. Another group has found, however, that a single glycine substitution in BPV1 E1 serine 48, which is phosphorylated by CK2, renders it a genome that is not able to replicate in C127 cells [41]. Taken together, conflicting evidence has been proposed for CK2 in regulating the activity of BPV replication proteins.

Our data show that the CK2 inhibitor CX4945 has a negative effect on E1 proteins encoded by HPV11 and HPV18 genomes. We demonstrate that the levels of the E1 proteins decrease markedly upon *CK2α* RNAi or CX4945 challenge. Moreover, the reduced level of E1 protein is not caused by transcriptional silencing or decreased copy number of the viral genome, since the level of the E1 protein was significantly reduced already after four hours of challenge with CX4945 and proteasome inhibitor MG132 alleviated CX4945-induced degradation of E1 protein. Thus far, the effect of CK2 on the stability of E1 protein has not been shown. Instead, cdk2-mediated phosphorylation has been shown to stabilize BPV E1 protein *in vitro* [42][43]. Analysis of the *in vivo* effect of cdk has shown the requirement of the kinase for nuclear retention of HPV E1 [14][15]. Therefore, our data provide for the first time a glimpse of how the stability of HPV E1 protein is regulated *in vivo* by CK2.

## Methods

### Plasmids

Parental plasmids encoding HPV5, HPV11 and HPV18 genomes on the basis of the pMC.BESPX minicircle production vector have been described previously [44][45][46]. The hemagglutinin (HA) epitope encoding sequence was inserted into the HPV11pMC.BESPX and HPV18pMC.BESPX parental plasmids in their E1 open reading frames (ORFs) after the 15^th^ nucleotide starting from the E1 1^st^ AUG. The resulting constructs were named HPV11E1HApMC.BESPX and HPV18E1HApMC.BESPX. The HPV18NLucpMC.BESPX construct was generated by cloning the codon optimized NLuc encoding sequence followed by the 2A region of the FMDV and the wt E2 encoding sequence after the 72^nd^ nucleotide of the HPV18 E2 ORF that corresponds to the E1 stop codon. Along with wt HPV18 proteins, the resulting construct contains NLuc fused with 24 amino acids of E2 protein N-terminally and self-processed FMDV-2A sequence C-terminally. The HPV11NLucpMC.BESPX and HPV5NLucpMC.BESPX constructs were generated using the same approach. All of the abovementioned HPV genomes were generated as minicircle plasmids in *E*. *coli* strain ZYCY10P3S2T using minicircle DNA technology as previously described [46]. The HPV16 and HPV31 genomes have been previously described [27]. Plasmids encoding N-terminally Flag-tagged CK2α and CK2α’ subunits were generated by cloning CK2α and CK2α’ ORFs lacking the 1^st^ ATG into the pCMV-Flag-4 vector (Sigma-Aldrich) between the HindIII and BamHI sites. Flag-tagged CK2α and CK2α’ kinase activity-deficient mutants CK2α(K68R) and CK2α’(K69R) bearing a point mutation in their ATP binding pockets at positions 68 and 69, respectively, were generated by PCR mutagenesis using CK2αpCMV-Flag-4 and CK2α’pCMV-Flag-4 templates and the following primers: CK2α(K68R) sense (s) GAAAAAGTTGTTGTTAGAATTCTCAAGCCAG, CK2α(K68R) antisense (as) CTGGCTTGAGAATTCTAACAACAACTTTTTC, CK2α’(K69R) s GAGAGAGTGGTTGTAAGAATCCTGAAGCC, CK2α’(K69R) as CTTCACTGGCTTCAGGATTCTTACAACCACTC. All new constructs were verified by DNA sequencing. All plasmid DNAs were purified from bacteria using the NucleoBond Xtra Midi EF Kit (Macherey-Nagel).

### Cell culture

The human osteosarcoma cell line U2OS (ATCC No HTB-96) was propagated in normal growth medium (NGM) containing Iscove's Modified Dulbecco's Medium (IMDM, Pan Biotech), 10% fetal calf serum (FCS) and 1% penicillin/streptomycin (PEST, Sigma-Aldrich) at 37°C and 5% CO_2_. Cells were transfected by electroporation (220 V and 975 μF) using a Gene Pulser XCell system (Bio-Rad Laboratories). The following amounts of wt or modified HPV minicircle genomes were used for transfection of 10^6^ U2OS cells: HPV5 1.5 μg, HPV11 0.3 μg, HPV18 1.2 μg. The HPV16 and HPV31 genomes were religated and transfected as previously described [27]. We transfected 250 and 125 ng of CK2αpCMV-Flag-4 and CK2α’pCMV-Flag-4 constructs, respectively, per 10^6^ U2OS cells. Transfected U2OS cells were seeded at an approximate density of 3x10^4^ per 1 cm^2^.

CIN612 cells and NHEKs (PromoCell) (kind gift from Dr. Frank Stubenrauch and Dr. Ana Rebane, respectively) were grown in Defined Keratinocyte-SFM Medium (DKSM) (Gibco, Thermo Fisher Scientific). Cells were detached using 0.25% Trypsin-EDTA solution, immediately transferred into DKSM containing 25% NGM, centrifuged at room temperature (RT) and 1400 rpm for 3 min and plated onto a new culture dish and fresh DKS after every 3–4 days. CIN612 cells and NHEKs were transfected with 1.5 μg of HPVNLuc minicircle genomes using 1.5 μl Plus reagent and 2.5 μl Lipofectamine LTX (Invitrogen, kind gift of Dr. Eva Žusinaite) per 12 wells of 96-well plate. 293T cells (ATCC no CRL-3216) were propagated in DMEM-high glycose (Sigma-Aldrich) supplemented with 10% FCS and 1% PEST. CX4945 was purchased from Santa Cruz Biotechnology and MG132 (kind gift of Dr. Reet Kurg) was purchased from Sigma-Aldrich. Both chemicals were diluted in DMSO.

### DNA isolation

Total and extrachromosomal DNA was extracted from cells as described previously [27]. DNA isolated from transfected U2OS cells was treated with DpnI restriction enzyme to digest bacterially methylated input DNA. The following restriction endonucleases were used to linearize HPV genomes: SacI (HPV5), HindIII (HPV11 and HPV31b), BglI and BstXI (HPV18 and its mutants), EcoRI (HPV31), and BamHI (HPV16).

### Southern blotting

Southern transfer and hybridization were performed as described [27]. The following amounts of total DNA were used for the detection of different HPV genomes: HPV5–5 μg; HPV11–2 μg; HPV18–5 μg; HPV31–10 μg; and HPV31b (CIN612)– 1.5 μg. HPV16 genome was detected using 30 μg of Hirt extract. All SB assays were performed at least three times, and representative images are shown. SB signals corresponding to the replicated HPV genomes were quantified using ImageQuant software.

### RNA interference

The following siRNAs were used: *CK2α* UGUCCGAGUUGCUUCCCGA 20 nM, *CK2α’* GCUGCGACUGAUAGAUUGG 30 nM, scrambled Neg. UAGCGACUAAACACAUCAA (Sigma-Aldrich). All siRNAs contained dTdT overhang. Initially, U2OS cells were transfected with siRNAs together with HPV genomes by electroporation. In the case of prolonged incubation, siRNAs were additionally delivered on the 3^rd^ day after the 1^st^ transfection using Lipofectamine RNAiMax reagent (Thermo Fisher Scientific) according to the manufacturer’s instructions.

### RNA isolation and PCR

Total RNA was isolated using the Quick RNA MiniPrep Kit (Zymo Research). Approximately 10 μg of total RNA was treated with 8 U of Turbo DNase (Thermo Fisher Scientific) for at least 4 h and subsequently precipitated with 7.5 M LiCl. Complementary DNA was synthesized using 1.2 μg of total RNA, oligo(dT) and RevertAid First Strand cDNA Synthesis Kit (Thermo Fisher Scientific) in the presence or absence of Reverse Transcriptase, if indicated. RT-PCR and quantitative RT-PCR (qPCR) were performed using HOT FIREPol PCR Mix and HOT FIREPol EvaGreen qPCR Mix (Solis Biodyne), respectively. The primers used are listed in S1 Table. The data of qPCR analyses are expressed as normalized with *ACTB* or *GAPDH* average means ± SD of three measurements obtained from at least three independent experiments.

### Luciferase assay and alkaline phosphatase activity

NLuc and FFLuc activities were measured using a Nano-Glo Dual-Luciferase Reporter Assay System (Promega) according to the manufacturer’s instructions for 96-well plates. Alkaline phosphatase activity and total protein concentrations were measured as previously described [47] and used for normalization of NLuc activity.

### Immunoprecipitation

U2OS cells were transfected with HPV11E1HA or HPV18E1HA minicircle genomes, incubated for the indicated periods of time, washed with PBS and lysed. WCEs of approximately 10^7^ cells were used for each immunoprecipitation (IP) of endogenous HA-tagged E1. WCEs were prepared in RIPA buffer (50 mM Tris pH 7.5, 150 mM NaCl, 2 mM EDTA, 0.1% SDS and 0.1% TRITON-X100) supplemented with protease inhibitor cocktail (PIC, Roche). Approximately 2x10^7^ cells were fractioned to cytoplasmic and nuclear lysates using NE-PER Nuclear and Cytoplasmic Extraction Reagents (Thermo Fisher Scientific). Cytoplasmic and nuclear extracts were diluted in 5 ml of RIPA buffer containing PIC. Lysates were incubated with m-anti-HA (Sigma-Aldrich) or r-a-HA (Labas Ltd.) antibodies (2 or 3 μg per sample, respectively) and protein A Sepharose at 4°C at slow rotation overnight. Immuno-complexes were washed 3 times with 5 ml of RIPA buffer at 4°C for 15 min, lysed in Laemmli sample buffer, denatured at 100°C for 5 min and subjected to SDS-PAGE analysis.

### Immunoblotting

WB was performed as previously described [48]. The following antibodies were used: m-a-HA (clone HA-7, Sigma-Aldrich) 1:3000, rat-a-HA-HRP (clone 3F10, Sigma-Aldrich) 1:1500, r-a-HA (Labas Ltd.) 1:2000, m-a-CK2α (Santa Cruz Biotechnologies) 1:300, m-a-CK2α’ (Santa Cruz Biotechnologies) 1:300, m-a-GAPDH (Sigma-Aldrich) 1:10000, m-a-lamin B (Santa Cruz Biotechnologies) 1:500, a-Flag-HRP M2 (Sigma-Aldrich) 1:5000, m-a-tubulin α (Sigma-Aldrich) 1:5000, m-a-myc (Sigma-Aldrich) 1:1000. The SuperSignal West Dura Extended Duration Substrate ECL Kit was used for the detection of E1-HA and CK2α proteins; other proteins were detected using the SuperSignal West Pico Kit (both Thermo Fisher Scientific). All immunoblotting assays were performed at least two times, and representative images are shown.

### Kinase activity assay

Flag-tagged CK2 catalytic subunits or their kinase activity-deficient mutants were overexpressed in 293T cells for 48 h and immuno-purified using Flag-M2 affinity binding resin (Sigma-Aldrich) according to the manufacturer’s instructions. Immuno-complexes were washed two times with ice-cold kinase buffer (50 mM Tris-HCl pH 7.4, 100 mM MgCl_2_, 20 μM ATP). Kinase reactions were carried out in the kinase buffer in the presence of 1 μCi [γ-32P]-ATP and 1 μg of casein at RT for 30 min, stopped by addition of Laemmli sample buffer, incubated at 100°C for 5 min and subjected to SDS-PAGE.

### Cell cycle and MTT analyses

Cell cycle profiles of the HPV18-transfected U2OS cells or CIN612 cells treated with DMSO or different concentrations of CX4945 were analyzed as previously described [7]. Viability of the cells in the presence of different concentrations of CX4945 was measured using MTT assay. The cells were grown on 96-well plates. MTT 0.5% solution was added directly to 100 μl of growth media, and the cells were incubated at 37°C and 5% CO_2_ for 2–3 h until purple precipitate was formed. The incubation media was aspirated; the cells were treated with 100 μl of DMSO and incubated on a shaker at RT for 30 min. Optical density was measured at 540 nm.

## Supporting information

S1 Fig A. U2OS cells were transfected with the plasmid encoding for myc-tagged CK2β. On the next day, the cells were treated with different concentrations of CX4945 or DMSO for additional 24 h. CK2β protein and its phosphorylated form CK2β^P^ were analyzed using WB and a-myc antibody. B, C. U2OS cells were treated as indicated for 2 or 6 days. Cell cycle profile was analyzed using propidium iodide by flow cytometry (LSR II from Becton Dickinson).
(TIF)

S2 Fig A—E. U2OS cells were transfected with different HPV genomes and siRNAs. On the next day, the cells were treated with 6 μM CX4945, if indicated. The cells were propagated for the indicated periods of time. Total DNA was isolated, digested with the restriction enzymes linearizing the respective HPV genomes and analyzed using SB. The signals corresponding to the replicated HPV genomes were quantified and set as 100% in the samples treated with Neg. siRNA (or DMSO in the case of HPV16) and propagated for 3 days. Data are presented as the average mean of at least 3 independent experiments +/- SD.
(TIF)

S3 Fig A. Maps of HPV5NLuc, HPV11NLuc and HPV18NLuc were generated using Clone software; LCR–long control region. Restriction enzymes linearizing the HPVNLuc genomes are indicated. B. U2OS cells were transfected with HPV18wt and HPV18NLuc genomes and propagated for 2, 3 and 4 days. Total DNA was extracted, digested with DpnI and BglI restriction enzymes and analyzed using SB. C. Linear regression of quantified HPV18NLuc replication signals and normalized NLuc activity obtained in the same samples. Signals of HPV18NLuc replication or normalized NLuc activity were set as 1 in the sample transfected with 250 ng of HPV18NLuc and incubated for 3 days. The average means of three experiments +/- SD are plotted. R and p values were calculated using GraphPad software. D. U2OS cells were transfected with the HPV18NLuc genome and siRNAs and incubated for 3 and 5 days. Levels of CK2α, CK2α’ and tubulin proteins were analyzed using WB.
(TIF)

S4 Fig A. CIN612 cells were transfected with the indicated siRNAs and incubated for 3 or 6 days. The levels of the mRNA expression of the respective genes were measured by qPCR using 2 different pairs of primers, normalized with *GAPDH* mRNA expression levels and set as 1 in the samples treated with DMSO for 3 days; ND–not detected (Ct values exceeded 37) B. CIN612 cells were treated as indicated for 3 or 6 days (left and right panels, respectively). Cell cycle profile was analyzed using propidium iodide by flow cytometry.
(TIF)

S5 Fig Nuclear E1 protein is rapidly degraded in response to CK2 inhibitor. A. Replication of the HPV11wt and HPV11E1HA genomes in U2OS cells treated with CX4945 or DMSO was analyzed using SB and total DNA digested with DpnI and HindIII restriction enzymes. B. U2OS cells were transfected with the HPV11E1HA genome. CX4945 was added 48 h after transfection. Cells were incubated for the indicated periods of time and fractionated for isolation of total DNA and WCEs. The level of the replicated HPV11E1HA genome was analyzed using SB. Levels of immunoprecipitated HA-tagged E1 protein were analyzed using WB. GAPDH was used as a loading control. C. U2OS cells were transfected with the HPV18 genome and siRNAs, if indicated. The cells were incubated for 2 days and treated with DMSO or 6 μM CX4945 for 24 h. Total RNA was extracted, treated with Turbo DNase and used for cDNA synthesis in the presence or absence of reverse transcriptase (+ RT or–RT, respectively). *E1*, *E2*, *E1^E4*, *E8^E2* and *GAPDH* transcripts were analyzed using RT-PCR (*GAPDH* for 22 cycles, other transcripts for 36 cycles). D. Cells were transfected with the HPV11E1HA genome, challenged with CX4945 after 3 days for 4, 8 or 12 h, detached using trypsin-EDTA and fractionated for nuclear (Nuc) and cytoplasmic (Cyt) extracts. Levels of CK2α, CK2α’, lamin B and GAPDH proteins were detected by immunoblotting. HA-tagged E1 protein was immuno-purified using r-a-HA antibody and analyzed using WB and m-a-HA antibody.
(TIF)

S1 Table List of primers used in the study. (DOCX)
