# Supplementary figures and images for: Activity of CK2α protein kinase is required for efficient replication of some HPV types
Source: PLoS Pathog. 2019 May 15;15(5):e1007788. doi: 10.1371/journal.ppat.1007788 (PMC6538197; doi:10.1371/journal.ppat.1007788)

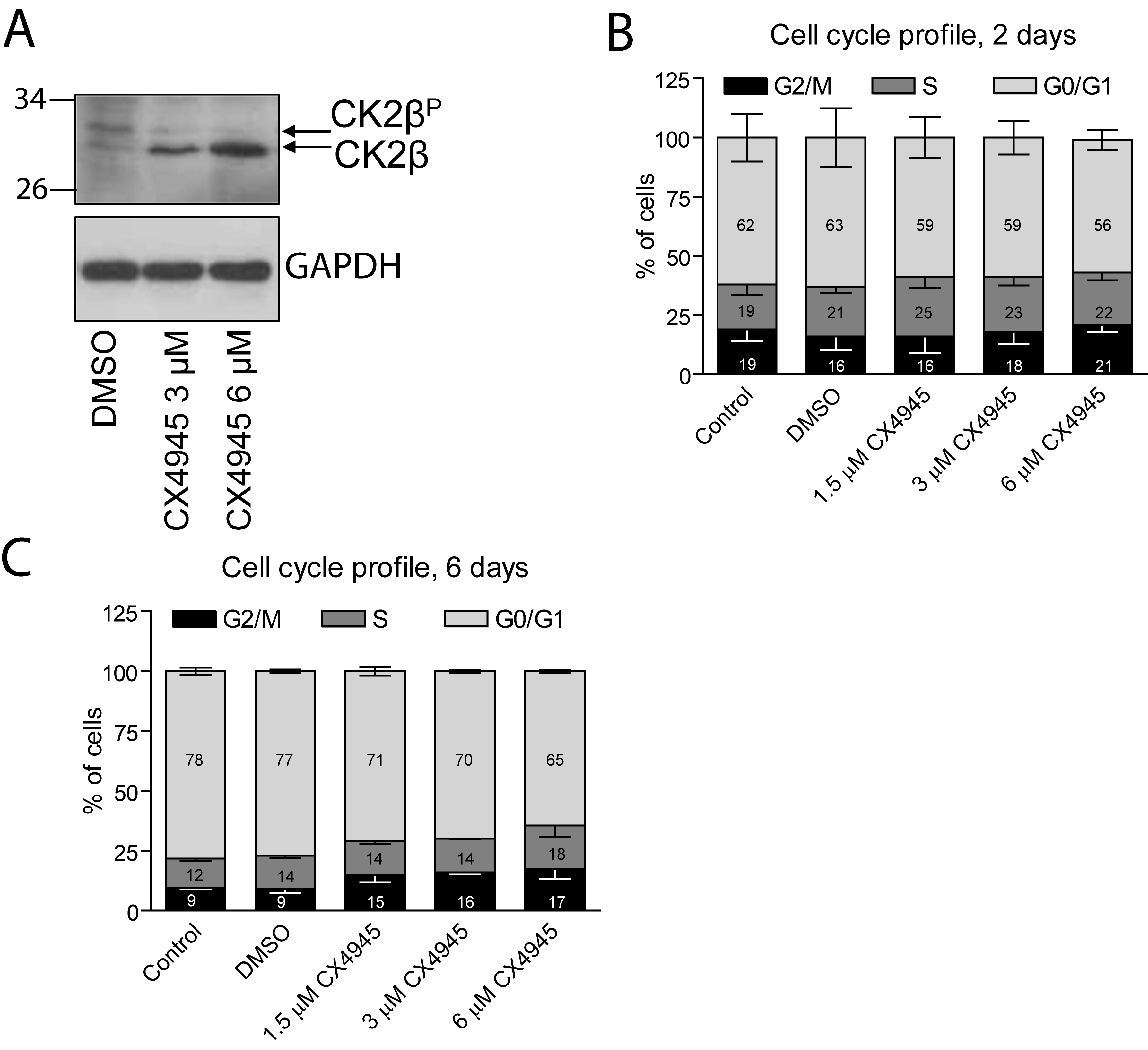

Supplement: S1 Fig — A. U2OS cells were transfected with the plasmid encoding for myc-tagged CK2β. On the next day, the cells were treated with different concentrations of CX4945 or DMSO for additional 24 h. CK2β protein and its phosphorylated form CK2βP were analyzed using WB and a-myc antibody. B, C. U2OS cells were treated as indicated for 2 or 6 days. Cell cycle profile was analyzed using propidium iodide by flow cytometry (LSR II from Becton Dickinson). (TIF) [file ppat.1007788.s001.tif]

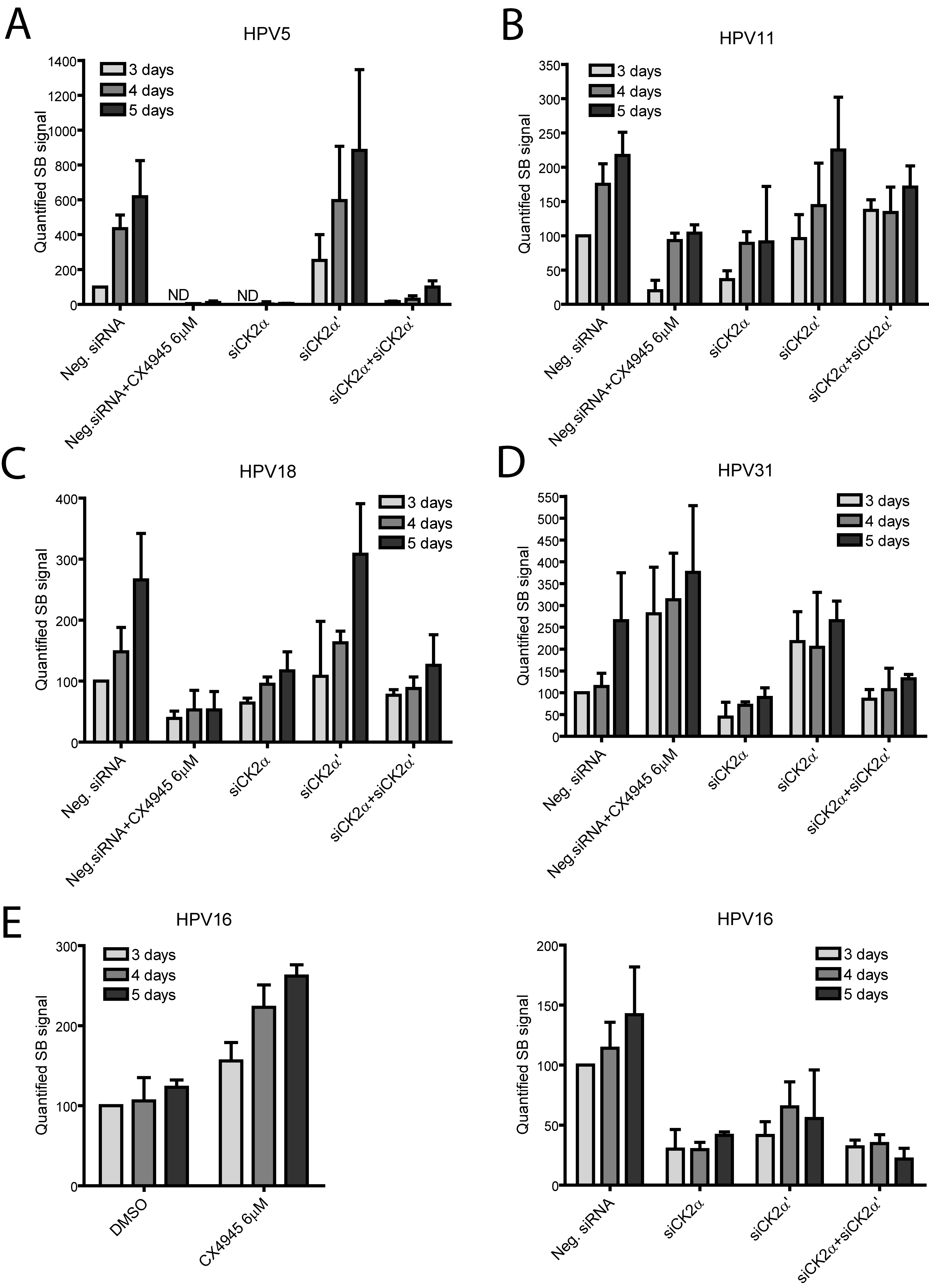

Supplement: S2 Fig — A—E. U2OS cells were transfected with different HPV genomes and siRNAs. On the next day, the cells were treated with 6 μM CX4945, if indicated. The cells were propagated for the indicated periods of time. Total DNA was isolated, digested with the restriction enzymes linearizing the respective HPV genomes and analyzed using SB. The signals corresponding to the replicated HPV genomes were quantified and set as 100% in the samples treated with Neg. siRNA (or DMSO in the case of HPV16) and propagated for 3 days. Data are presented as the average mean of at least 3 independent experiments +/- SD. (TIF) [file ppat.1007788.s002.tif]

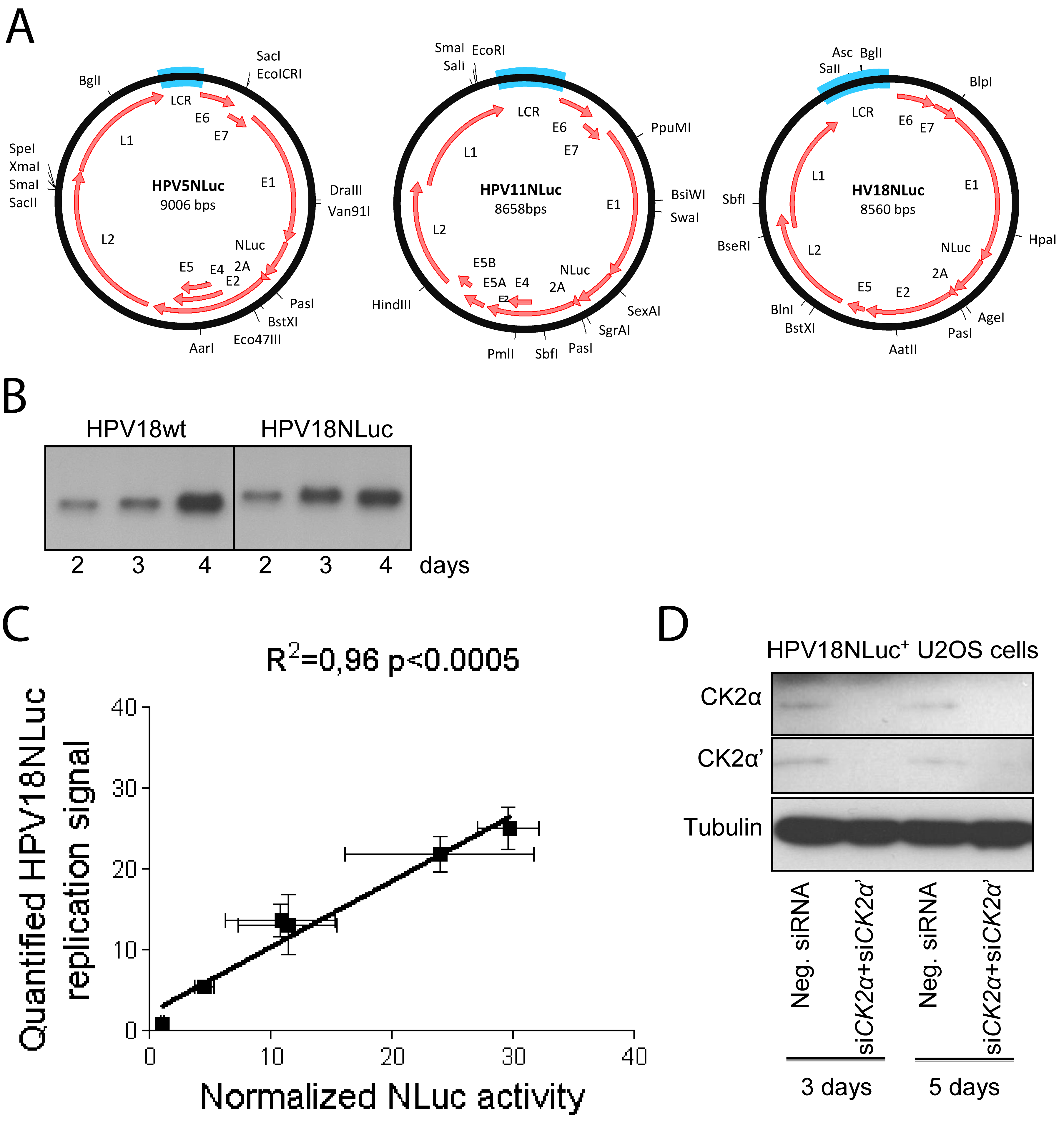

Supplement: S3 Fig — A. Maps of HPV5NLuc, HPV11NLuc and HPV18NLuc were generated using Clone software; LCR–long control region. Restriction enzymes linearizing the HPVNLuc genomes are indicated. B. U2OS cells were transfected with HPV18wt and HPV18NLuc genomes and propagated for 2, 3 and 4 days. Total DNA was extracted, digested with DpnI and BglI restriction enzymes and analyzed using SB. C. Linear regression of quantified HPV18NLuc replication signals and normalized NLuc activity obtained in the same samples. Signals of HPV18NLuc replication or normalized NLuc activity were set as 1 in the sample transfected with 250 ng of HPV18NLuc and incubated for 3 days. The average means of three experiments +/- SD are plotted. R and p values were calculated using GraphPad software. D. U2OS cells were transfected with the HPV18NLuc genome and siRNAs and incubated for 3 and 5 days. Levels of CK2α, CK2α’ and tubulin proteins were analyzed using WB. (TIF) [file ppat.1007788.s003.tif]

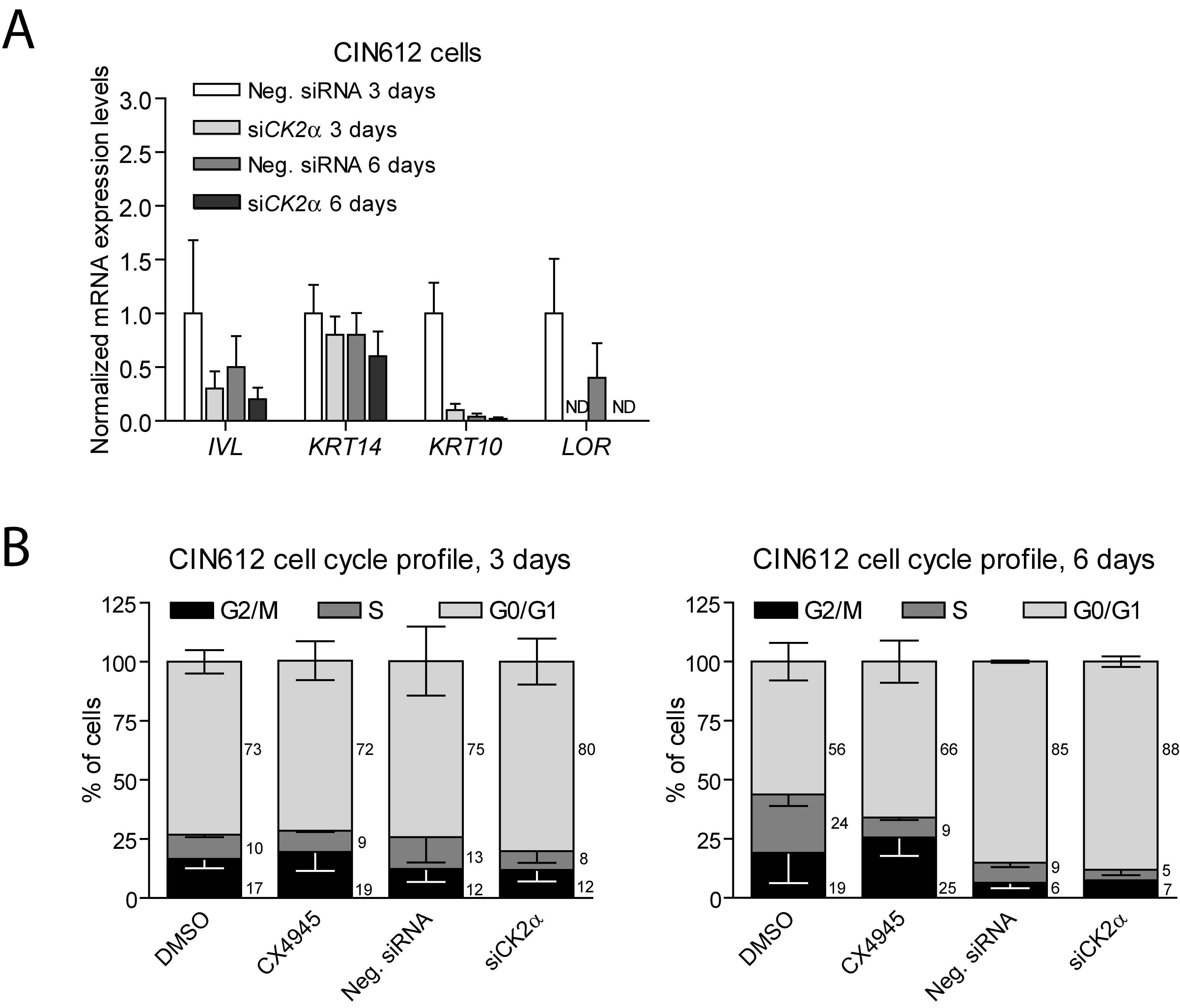

Supplement: S4 Fig — A. CIN612 cells were transfected with the indicated siRNAs and incubated for 3 or 6 days. The levels of the mRNA expression of the respective genes were measured by qPCR using 2 different pairs of primers, normalized with GAPDH mRNA expression levels and set as 1 in the samples treated with DMSO for 3 days; ND–not detected (Ct values exceeded 37) B. CIN612 cells were treated as indicated for 3 or 6 days (left and right panels, respectively). Cell cycle profile was analyzed using propidium iodide by flow cytometry. (TIF) [file ppat.1007788.s004.tif]

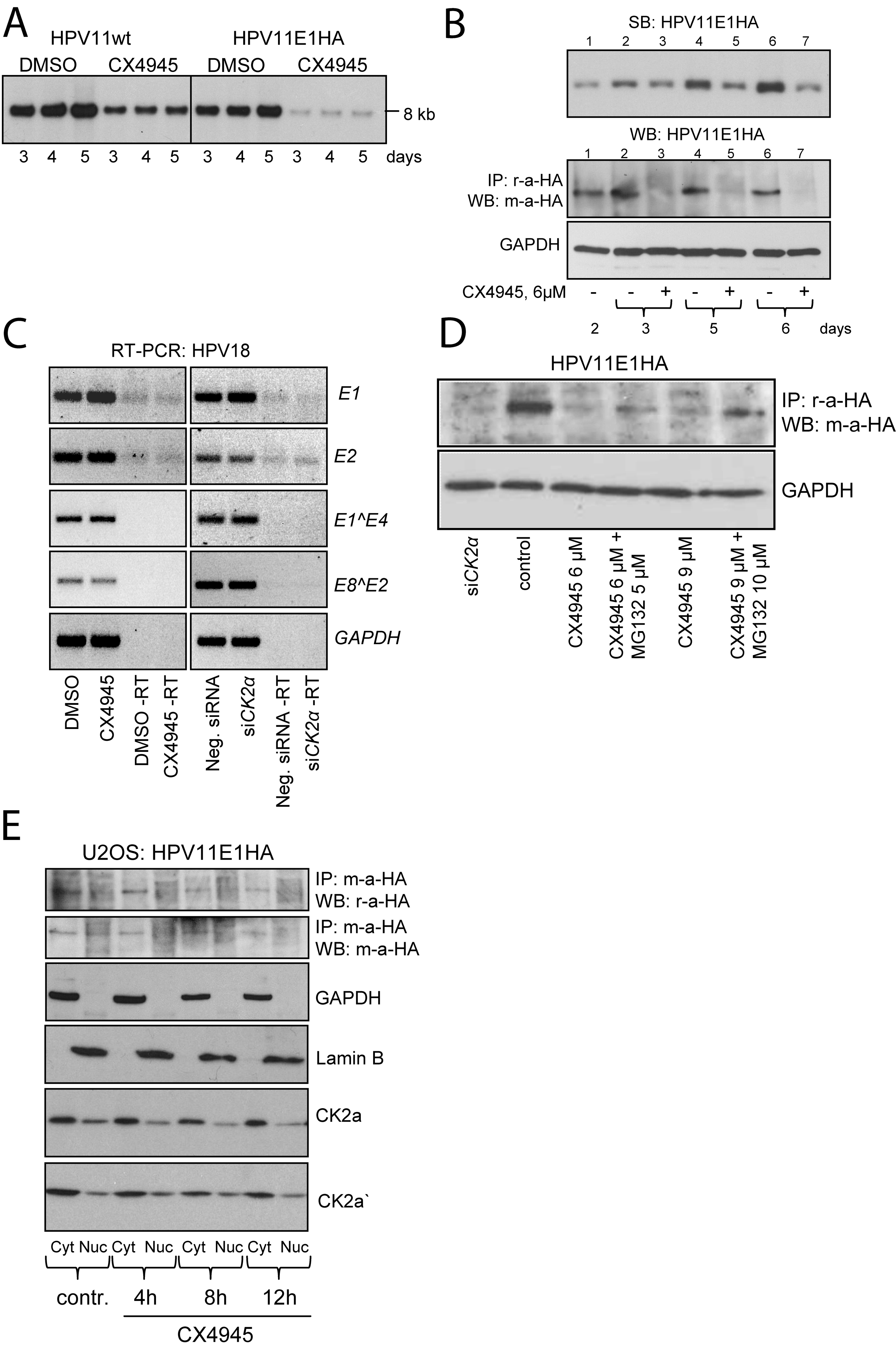

Supplement: S5 Fig — A. Replication of the HPV11wt and HPV11E1HA genomes in U2OS cells treated with CX4945 or DMSO was analyzed using SB and total DNA digested with DpnI and HindIII restriction enzymes. B. U2OS cells were transfected with the HPV11E1HA genome. CX4945 was added 48 h after transfection. Cells were incubated for the indicated periods of time and fractionated for isolation of total DNA and WCEs. The level of the replicated HPV11E1HA genome was analyzed using SB. Levels of immunoprecipitated HA-tagged E1 protein were analyzed using WB. GAPDH was used as a loading control. C. U2OS cells were transfected with the HPV18 genome and siRNAs, if indicated. The cells were incubated for 2 days and treated with DMSO or 6 μM CX4945 for 24 h. Total RNA was extracted, treated with Turbo DNase and used for cDNA synthesis in the presence or absence of reverse transcriptase (+ RT or–RT, respectively). E1, E2, E1^E4, E8^E2 and GAPDH transcripts were analyzed using RT-PCR (GAPDH for 22 cycles, other transcripts for 36 cycles). D. Cells were transfected with the HPV11E1HA genome, challenged with CX4945 after 3 days for 4, 8 or 12 h, detached using trypsin-EDTA and fractionated for nuclear (Nuc) and cytoplasmic (Cyt) extracts. Levels of CK2α, CK2α’, lamin B and GAPDH proteins were detected by immunoblotting. HA-tagged E1 protein was immuno-purified using r-a-HA antibody and analyzed using WB and m-a-HA antibody. (TIF) [file ppat.1007788.s005.tif]
