# Supplementary material for: Activity of CK2α protein kinase is required for efficient replication of some HPV types
Source: PLoS Pathog. 2019 May 15;15(5):e1007788. doi: 10.1371/journal.ppat.1007788 (PMC6538197; doi:10.1371/journal.ppat.1007788)
Supplement: S1 Table — (DOCX) [file ppat.1007788.s006.docx]

S1 Table. List of primers used in the study

| *GAPDH* | sense | CTCTCTGCTCCTCCTGTTCGAC |
| --- | --- | --- |
|  | antisense | TGAGCGATGTGGCTCGGCT |
| *ACTB* | sense | CAGAGCCTCGCCTTTGCCGA |
|  | antisense | ATCCATGGTGAGCTGGCGGC |
| *CK2α* | sense | GAGATGTCAAGCCCCATAATGTC |
|  | antisense | GAAGTATCGGGAAGCAACTCGG |
| *CK2α'* | sense | CACAGGGATGTGAAACCTCAC |
|  | antisense | CCTTGAGGCTACACGAACATTG |
| *HPV18 E1* | sense | CATTTACCAGCCCGACGAG |
|  | antisense | AAACCAGCCGTTACAACCCG |
| *HPV18 E2* | sense | GATAGTGGCTATGGCTGTTC |
|  | antisense | GCTGTTGTTGCCCTCTGTG |
| HPV18 *E1^E4* | sense | CATTTACCAGCCCGACGAG |
|  | antisense | GACGTCTGGCCGTAGGTCTTTGC |
| HPV18 *E8^E2* | sense | GATAGTGGCTATGGCTGTTC |
|  | antisense | GACGTCTGGCCGTAGGTCTTTGC |
| *K14* | sense | GAAGTGAAGATCCGTGACTG |
|  | antisense | GACATTGGCATTGTCCACTG |
| *K14* | sense | CATGAGTGTGGAAGCCGACAT |
|  | antisense | GCCTCTCAGGGCATTCATCTC |
| *IVL* | sense | TTCCTCCTCCAGTCAATACC |
|  | antisense | CTCAGGCAGTCCCTTTACAG |
| *IVL* | sense | TCCTCCAGTCAATACCCATCAG |
|  | antisense | GCAGTCATGTGCTTTTCCTCTTG |
| *LOR* | sense | CTACCTGGCCGTCCAAATAG |
|  | antisense | CTCGGGTAGCATCATGAGAG |
| *LOR* | sense | CACCCTTCCTGGTGCTTTG |
|  | antisense | AGAGGTCTTCACGCAGTCC |
| *K10* | sense | TTGCTGAACAAAACCGCAAAG |
|  | antisense | GCCAGTTGGGACTGTAGTTCT |
| *K10* | sense | GGGCTCTGGAAGAATCAAAC |
|  | antisense | CTGGCATTGTCGATCTGAAG |
